# Lifestyle and Non-muscle Invasive Bladder Cancer Recurrence, Progression, and Mortality: Available Research and Future Directions

**DOI:** 10.3233/blc-190249

**Published:** 2020-03-28

**Authors:** Kyle B. Zuniga, Rebecca E. Graff, David B. Feiger, Maxwell V. Meng, Sima P. Porten, Stacey A. Kenfield

**Affiliations:** a Department of Urology, University of California, San Francisco, CA, USA; b Osher Center for Integrative Medicine, University of California, San Francisco, CA, USA; c College of Physicians and Surgeons, Columbia University Medical Center, New York, NY, USA; d Department of Epidemiology and Biostatistics, University of California, San Francisco, CA, USA; e School of Medicine, Duke University Medical Center, Durham, NC, USA; f Department of Emergency Medicine, Northwestern University Feinberg School of Medicine, Chicago, IL, USA

**Keywords:** Urinary bladder neoplasms, lifestyle, body mass index, smoking, diet, diabetes mellitus, dietary supplements, prognosis, recurrence, non-invasive

## Abstract

**BACKGROUND:**

A broad, comprehensive review of studies exploring associations between lifestyle factors and non-muscle invasive bladder cancer (NMIBC) outcomes is warranted to consolidate recommendations and identify gaps in research.

**OBJECTIVE:**

To summarize the literature on associations between lifestyle factors and clinical outcomes among patients with NMIBC.

**METHODS:**

PubMed was systematically queried for articles published through March 2019 regarding lifestyle factors and recurrence, progression, cancer-specific mortality, and all-cause mortality among patients with NMIBC.

**RESULTS:**

Notwithstanding many ambiguities, there is good-quality evidence suggesting a benefit of smoking avoidance/cessation, healthy body mass index (BMI), and type II diabetes mellitus prevention and treatment. *Lactobacillus casei* probiotic supplementation may reduce recurrence. There have been individual studies suggesting a benefit for uncooked broccoli and supplemental vitamin E as well as avoidance of supplemental vitamin B9, areca nut chewing, and a “Western diet” pattern high in fried foods and red meat. Additional studies do not suggest associations between NMIBC outcomes and use of fibrin clot inhibitors; insulin and other oral hypoglycemics; statins; supplemental selenium, vitamin A, vitamin C, and vitamin B6; fluid intake and intake of specific beverages (e.g., alcohol, coffee, green tea, cola); various dietary patterns (e.g., Tex-Mex, high fruit and vegetable, low-fat); and occupational and chemical exposures.

**CONCLUSIONS:**

Despite a myriad of publications on lifestyle factors and NMIBC, a need remains for research on unexplored associations (e.g., physical activity) and further studies that can elucidate causal effects. This would inform future implementation strategies for healthy lifestyle change in NMIBC patients.

## INTRODUCTION

Bladder cancer is the 2nd most common cancer of the genitourinary system [1]. In the United States (US) alone, roughly 80,000 new cases and 18,000 deaths from bladder cancer are expected in 2019 [1]. Non-muscle invasive bladder cancer (NMIBC) comprises approximately 70% of new cases [2]. Bladder cancer-specific survival of high-grade NMIBC ranges from 70–85% at ten years, and rates are even higher among those with low-grade disease [3, 4]. Still, the five-year probability of recurrence and progression ranges from 31% to 78% and from less than 1% to 45%, respectively, depending on clinical and pathological severity [5]. Given the chronicity of NMIBC, it is essential to elucidate lifestyle factors that may reduce the risk of poor outcomes.

Previous systematic reviews and meta-analyses have consolidated available data regarding associations between lifestyle factors and NMIBC prognosis. For example, one meta-analysis reported an increased risk of recurrence and bladder cancer-specific mortality (CSM) among current and former smokers [6]. Another reported an increased risk of recurrence among obese and overweight patients [7]. The latter review also included a discussion of dietary factors and supplements. However, given the rapidly expanding literature on a growing number of lifestyle factors that may influence NMIBC outcomes, an updated review summarizing a wider variety of lifestyle factors is warranted. To this end, we aimed to compile a comprehensive overview of studies evaluating associations between lifestyle factors and recurrence, progression, CSM, and all-cause mortality (ACM) in patients with NMIBC.

## MATERIALS AND METHODS

### Evidence acquisition

**Fig. 1.**
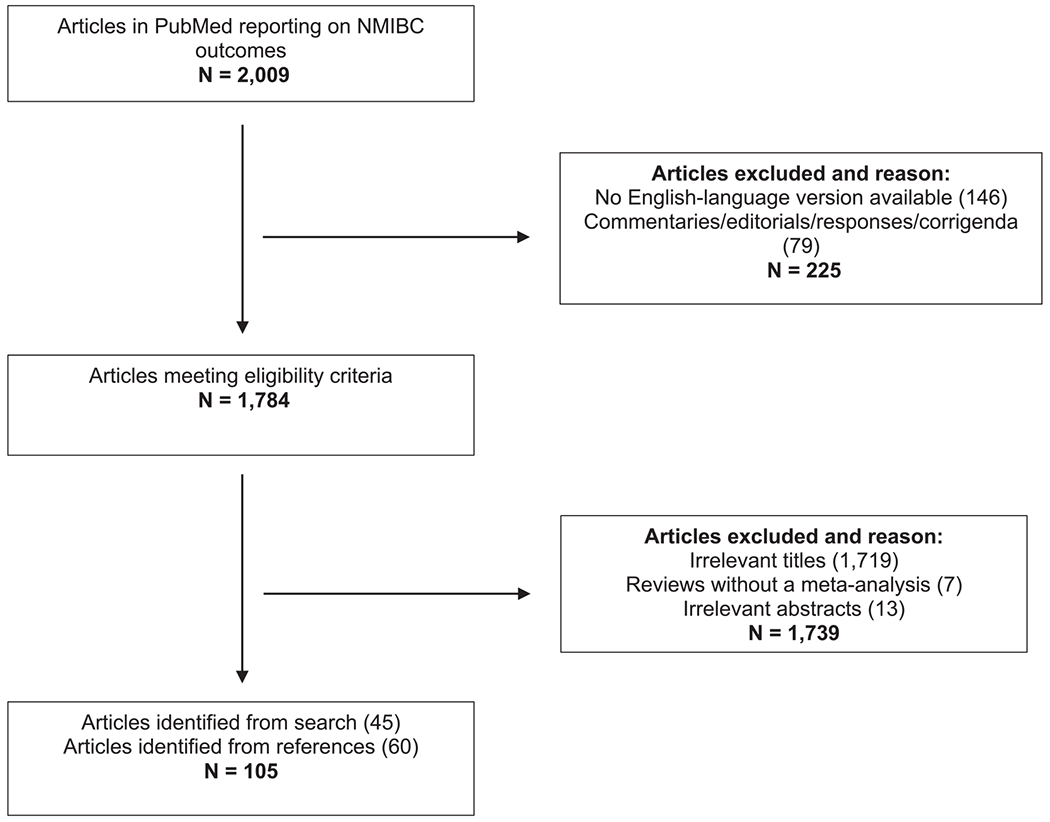
Flow diagram showing article identification, eligibility, and inclusion.

We sought to include all studies that quantitatively evaluated associations between lifestyle factors and NMIBC outcomes. In March 2019, a PubMed literature search was conducted based on the following terms: (cancer OR carcinoma OR neoplas* OR tumor) AND (bladder OR urothelial OR “transitional cell”) AND (NMIBC OR non-muscle-invasive) AND (“risk factor” OR recur* OR progression OR death OR survival OR fatal OR prognos* OR outcome). The result was 2,009 articles for review. Articles were considered relevant if they: included participants with a diagnosis of NMIBC; assessed at least one lifestyle factor; and did so in relation to recurrence (reappearance of tumor following initial treatment), progression (upstaging to muscle-invasive, nodal, and/or metastatic disease and cystectomy for treatment-resistant disease), CSM, and/or ACM. Upon selection of 45 relevant articles, we reviewed the reference sections to identify additional relevant studies. We ultimately identified a total of 105 articles published between 1977–2019 for inclusion. Full article screening and inclusion criteria are outlined in Fig. 1. Study inclusion was consistent with Preferred Reporting Items for Systematic Reviews and Meta-Analysis (PRISMA) guidelines [8]. As a literature review, this study is exempt from any requirement for Institutional Review Board approval.

### Evidence synthesis

Table 1 summarizes lifestyle factors with evidence of benefit relative to NMIBC outcomes. Supplementary Table 1 details the design and results of each included study.

## RESULTS

### Smoking

Fifty-seven studies have assessed associations between smoking-related exposures and NMIBC outcomes [6, 9–64]. Multiple exposure types have been studied, including current versus never, [6, 9–59] former versus never, [6, 9–36, 60] current versus former, [9–15, 60–63] and cumulative exposure (e.g., pack-years, quantity/day, cigarette index) [9–13, 16–19, 37, 38, 60, 64].

The sum of existing evidence suggests that smoking may be moderately associated with recurrence [6, 9–12, 14, 16–35, 37, 39–53, 60–62, 64]. One meta-analysis of 11 studies of 6,908 NMIBC patients reported an increased risk of recurrence among current smokers (at the time of questionnaire completion, interview, or from patient record review) compared to nonsmokers (hazard ratio [HR] 1.27, 95% confidence interval [CI] 1.09–1.46). The association for former smokers relative to nonsmokers was attenuated (n = 5,382 NMIBC patients, HR 1.13, 95% CI 1.00–1.25) [20]. Another meta-analysis of 15 studies that included 10,192 patients with NMIBC or MIBC reported an increased risk of recurrence in both current (summary relative risk estimate [SSRE] 1.23, 95% CI 1.05–1.45) and former smokers (SSRE 1.22, 95% CI 1.09–1.37) relative to never smokers. The meta-analysis did not, however, find an association with progression among 5 studies with 3,979 NMIBC or MIBC patients [6]. Indeed, the evidence regarding smoking and disease progression has been ambiguous [6, 9–11, 13, 15, 16, 18–24, 39–46, 60, 61]. The majority of studies among patients with NMIBC alone have reported no association between smoking and CSM [13, 15, 20, 25, 39–41, 54] or ACM [11, 25, 26, 39, 40]. However, in studies with a mix of patients with NMIBC and MIBC, the evidence regarding an association between smoking and CSM [6, 18, 21, 36, 42, 43, 55, 63] or ACM [18, 21, 36, 38, 42, 43, 56–59, 63] has been mixed. In the latter aforementioned meta-analysis, the authors also reported an increased pooled risk of CSM for both current (SRRE 1.28, 95% CI 1.07–1.52) and former smokers (SRRE 1.20, 95% CI 1.03–1.41) in 5 studies with 4,372 patients with NMIBC and MIBC [6].

Regarding cumulative exposure, Rink et al. reported a substantially reduced risk of recurrence and progression among short-term (≤19.9 years) smokers and a reduced risk of progression among light (≤19 cigarettes per day) long-term smokers compared to long-term heavy smokers, albeit no difference in ACM [10]. Still, a case-control study by Leibovici et al. of 519 patients with NMIBC and 505 healthy controls examined pack-years as a continuous variable and reported no association with recurrence or progression [19].

Overall, compared to current smoking, long-term smoking cessation may be associated with improved outcomes compared to current smoking [9–11, 37, 38, 60, 61]. In an international retrospective cohort of 2,043 patients with NMIBC, Rink et al. explored the association of multiple types of smoking exposures on recurrence, progression, and ACM [10]. Compared to current smokers, patients who quit smoking ≥ 10 years prior to NMIBC diagnosis had a reduced risk of recurrence (HR 0.66, 95% CI 0.52–0.84) and progression (HR 0.42, 95% CI 0.22–0.83), albeit no difference in ACM. In contrast, a prospective cohort of 722 patients with NMIBC showed no difference in risk of recurrence in those who had quit smoking greater than 40 years prior to diagnosis and current smokers [12].

### Body mass index

Eighteen studies have assessed the association between body mass index (BMI) and prognosis [7, 24, 25, 30, 34, 39, 59, 65–75]. One study assessed BMI at diagnosis, [24] 13 studies assessed BMI at time of treatment (e.g., transurethral resection of bladder tumor (TURBT), radical cystectomy), [25, 30, 34, 39, 59, 65–72] and three studies did not report timing of BMI measurement [73–75].

Among NMIBC patients, the majority of studies suggest that elevated BMI is associated with increased risks of recurrence [7, 24, 34, 65, 66, 73, 74] and progression [65, 66, 73, 74]. The association between BMI and recurrence is more conflicting in studies with both NMIBC and MIBC patients [7, 25, 30, 68, 70]. In a meta-analysis of three studies of 1,633 patients with NMIBC, Westhoff et al. reported that being overweight (BMI 25–29.9 kg/m^2^; pooled HR 1.29, 95% CI 1.05–1.58) or obese (BMI ≥ 30 kg/m^2^; pooled HR 1.82, 95% CI 1.12–2.95) was associated with recurrence [7]. Westhoff et al. also meta-analyzed the results of three studies among patients with NMIBC and MIBC and reported no association between BMI and recurrence. Although Westhoff et al. reported no association with progression, [7] multiple studies have suggested an increased risk [65, 66, 73, 74]. Lenis et al. investigated the association between metabolic syndrome (MetS) and outcomes in patients with NMIBC undergoing TURBT. MetS was not associated with recurrence or progression overall, but analyses of MetS components indicated that obesity was a strong predictor (HR 3.42, 95% CI 1.55–7.52) [73]. Wyszynski et al. reported that smoking may work synergistically with BMI to worsen outcomes among patients with NMIBC; [24] while being overweight was not associated with recurrence among never or former smokers, it was statistically significantly associated with recurrence risk among current smokers (HR = 2.24, 95% CI 1.15–4.34).

The association between BMI and CSM and ACM among patients with NMIBC remains unclear [39, 65, 74]. In studies among those with NMIBC and MIBC, studies of mortality outcomes primarily vary between no association [7, 25, 59, 67, 69, 71] and increased mortality risk [25, 30, 68, 70]. The largest study observing the association between BMI and outcomes consisted of 4,118 patients with NMIBC and MIBC treated with radical cystectomy. Multivariable analyses demonstrated an increased risk of both CSM (HR 1.43, 95% CI 1.24–1.66) and ACM (HR 1.81, 95% CI 1.60–2.05) comparing obese patients to those with BMI <25 kg/m^2^ [68]. In contrast, the meta-analysis by Westhoff et al. and a prospective cohort study by Gierth et al. reported no association between BMI and CSM [7, 75]. Gierth et al. did, however, report reduced ACM among obese patients (HR 0.60, 95% CI 0.39–0.92) compared to those with healthy weight [75].

Two studies hypothesized that sarcopenia rather than BMI is a better predictor of outcomes in patients undergoing cystectomy [59, 71]. Psutka et al. used computerized tomography to calculate fat mass index and skeletal muscle index among patients with NMIBC and MIBC. In models including BMI and sarcopenia, they found no association between BMI and ACM, while sarcopenia was associated with an increased risk of ACM (HR 1.67, 95% CI 1.11–2.50). Furthermore, in a sub-analysis of nonsarcopenic patients, the authors reported a 7% decreased risk of ACM for every 1 kg/m^2^ increase in BMI (*p* = 0.008) [59]. Future directions for studies on sarcopenia include examining its association with outcomes among patients with lower risk disease undergoing surveillance.

### Diabetes and diabetic drugs

Eleven studies have investigated the association between type II diabetes mellitus (DMII) and/or diabetic drugs and NMIBC outcomes [25, 29, 40, 44, 45, 66, 72, 73, 76–78].

Among patients with NMIBC, there have been mixed reports on associations between DMII and both recurrence [29, 40, 44, 45, 66, 72, 73] and progression [40, 44, 45, 66, 72, 73]. Based on retrospective data on 1,117 patients with NMIBC, Reiken et al. reported that those with DMII not treated with metformin had a statistically significantly increased risk of recurrence (HR 1.39, 95% CI 1.04–1.86) and progression (HR 2.21, 95% CI 1.29–3.77) compared to those without DMII [40]. Those with DMII treated with metformin had a reduce risk of recurrence compared to those without DMII (HR 0.48, 95% CI 0.26–0.89). In regard to glycemic control, Ahn et al. reported that a higher proportion of those with HbA1c ≥ 7.0% experienced progression compared to those with HbA1c < 7.0% (28.8% versus 11.5%, *p* = 0.026). However, this association became non-significant upon multivariable analysis [72]. Studies among NMIBC patients have reported a null association between DMII and CSM [40, 76, 78]. However, a study of 13,811 patients with NMIBC aged 66 years or older reported DMII was associated with a slightly increased risk of ACM (HR 1.04, 95% CI 1.03–1.05) compared to no DMII [76]. The use of metformin, insulin, and other hypoglycemics does not appear to be associated with CSM or ACM, [40, 78] and glyburide may be associated with an increased risk of CSM [78].

In a study of NMIBC and MIBC patients undergoing radical cystectomy, DMII treated with metformin was not associated with recurrence, CSM, or ACM compared to no DMII. Patients with DMII not treated with metformin, however, did have an increased risk of CSM (HR 1.53, 95% CI 1.12–2.09) and ACM (HR 1.52, 95% CI 1.16–2.00) [25]. Nayan et al. reported that among patients with DMII, metformin use may be associated with a reduced risk of recurrence (HR 0.38, 95% CI 0.20–0.72) and CSM (HR = 0.57, 95% CI 0.35–0.91) [77]. Insulin and other hypoglycemic agents did not confer the same benefit.

### Drugs for cardiovascular health

Eighteen studies have reported on associations between drugs for cardiovascular health and NMIBC outcomes [30, 32, 33, 39, 41, 76, 79–90]. Among them, 10 have investigated statins, [30, 33, 39, 41, 76, 79–83] and 10 have investigated fibrin clot inhibitors (FCIs) like aspirin, warfarin, and celecoxib [32, 33, 39, 84–90].

A study of 84 patients with NMIBC by Hoffman et al. raised concern that statins may be associated with increased odds of progression and cystectomy [81]. However, this early analysis was based on a small cohort of patients, among whom only 19 took statins during intravesical bacillus Calmette-Guérin (BCG) therapy. Most subsequent studies have not replicated these findings, with the vast majority reporting no association between statin use and recurrence, [30, 39, 41, 79–83] progression, [39, 41, 79, 80, 82, 83] CSM, [30, 39, 41, 76, 80, 83] or ACM [39, 80, 82, 83]. Furthermore, Richard et al. reported a potential reduced risk of ACM with statin use [76]. In their retrospective review of the records of 13,811 older patients (median age 76) with NMIBC, they analyzed the association between cumulative statin exposure (rather than current use at diagnosis) and both CSM and ACM. After a median 7.1 years of follow-up, there was no improvement in CSM, but there was a slight reduction in ACM according to increased length of use (HR 0.93 per year of use, 95% CI 0.91–0.96).

As with statin use, two early studies found positive associations between FCIs and risk of NMIBC recurrence. However, one study only had 45 patients, [88] neither study included multivariable analyses, [85, 88] and they did not distinguish between types of FCIs. Many studies since have reported null associations between most FCIs and recurrence, [33, 39, 84, 86, 87, 89, 90] progression, [32, 39, 79, 84, 86, 87] CSM, [39, 87] or ACM [39, 86, 87]. Two studies have demonstrated an association between aspirin use and a reduced risk of recurrence [32] and progression to cystectomy [84]. For example, in a retrospective cohort of 907 patients with NMIBC or MIBC, Boorjian et al. reported that there was a slight reduction in progression to cystectomy among those using aspirin at time of BCG (HR 0.70, 95% CI 0.52–0.96) [84]. Despite these results, the majority of studies have reported no association with aspirin and NMIBC outcomes [33, 39, 87, 90]. Regarding warfarin use, Boorjian et al. reported an association with an increased risk of progression to surgery (HR 1.89, 95% CI 1.31–2.74) [84]. The effect of celecoxib was tested in two randomized controlled trials (RCTs) among patients with NMIBC undergoing BCG therapy [86, 89]. Both reported no association with recurrence. The BOXIT trial also reported no effect on progression to invasive disease or mortality, but did report suggestive increased incidence of serious cardiovascular events in the celecoxib group (5.2%) compared to the placebo group (1.7%, *p* = 0.07) [86].

### Diet and supplements

Twenty-five studies have reported on associations between diet and/or supplements and NMIBC outcomes [15, 29, 31, 47, 51, 52, 58, 91–108]. Five evaluated dietary patterns or individual food items, [29, 58, 94–96] five evaluated beverages, [15, 31, 47, 58, 97] 14 evaluated vitamins and minerals, [51, 91–94, 98–106] and three evaluated probiotics [52, 107, 108].

One retrospective cohort study of 239 patients with NMIBC assessed self-reported intake of multiple dietary items and CSM and ACM [95]. An intake of ≥ 1 serving of uncooked broccoli/month (mean 3.9 servings/month) was associated with a reduced risk of CSM (HR 0.43, 95% CI 0.25–0.74) and ACM (HR 0.57, 95% CI 0.39–0.83) compared to an intake of < 1 serving/month. This and two other studies reported no associations between fruit and/or vegetable consumption and recurrence, [94, 96] progression, [96] or mortality [95]. Another study reported that heavy areca nut chewing (>10 nuts/day), a practice common among individuals in South Asia, may be associated with an increased risk of recurrence (HR 2.18, 95% CI 1.37–3.47) compared to non-chewing [29].

Westhoff et al. grouped individual dietary items in order to observe associations of four dietary “patterns” with recurrence and progression in 595 patients with NMIBC [96]. The fruit and vegetable pattern, low-fat pattern (low-fat alternative foods like light salad dressing, low-fat dairy, etc.), and Tex-Mex pattern (Mexican food, barbecue, pizza) were not significantly associated with the outcomes of interest. However, the Western pattern, which consisted primarily of fried foods and red meat, was associated with an increased risk of recurrence (HR 1.48, 95% CI 1.06–2.06). The study did not investigate the individual dietary components that resulted in increased risk (e.g., red meat may confer a greater risk than fried foods or vice versa).

Two studies have reported a null association between total fluid intake and recurrence [31, 97]. Evidence leans toward no association between alcohol intake and NMIBC prognosis [15, 97]. Still, it is interesting to note that, among Japanese men with NMIBC or MIBC, Wakai et al. reported that moderate drinkers of < 2 gou/day (1 gou = 180 ml of Japanese sake) had a reduced risk of ACM (HR 0.41, 95% CI 0.22–0.77) compared to never drinkers [58]. Limited evidence suggests other beverages, such as coffee, green tea, or cola, and artificial sweetener do not appear to be associated with outcomes among patients with NMIBC [47, 58].

Among supplemental vitamins and minerals, one RCT reported a reduced risk of recurrence among those taking 400IU of supplemental vitamin E (RR 0.53, 95% CI 0.11–0.94) [100]. However, the sample was relatively small *(n* = 46) and the non-intervention group was not placebo-controlled. Another study reported that synthetic vitamin B9 (folate) may be associated with increased risk of recurrence (HR 1.80 in tertile 3 compared to tertile 1, 95% CI 1.14–2.84) [101]. Evidence leans toward questionable to no benefit from supplemental vitamin A, [51, 102–106] vitamin B6, [91, 92] selenium, [93] and megadose multivitamin formulations [98, 99]. Multiple RCTs have investigated the effect of synthetic retinoids like fenretinide [102, 103] and etretinate on NMIBC outcomes [104–106]. Effects on recurrence ranging from null [102–104, 106] to beneficial [105] have been reported, and high rates of ophthalmologic and dermatologic adverse effects may outweigh any indeterminate benefit for bladder cancer prognosis.

Finally, three RCTs have assessed the effect of probiotics containing Lactobacillus casei on prognosis. All reported a reduction in recurrence in the intervention group [52, 107, 108]. The largest trial with longest period of follow-up reported that, among 202 patients with NMIBC randomized to weekly intravesical epirubicin instillation with or without a daily oral probiotic supplement, those in the probiotic group had improved 3-year recurrence-free survival (74.6% versus 59.5%, *p* = 0.0234) [52]. Recurrence-free survival remained significant upon adjustment for tumor multiplicity, size, and stage (HR 0.57, 95% CI 0.35–0.93). However, there was no difference in progression, CSM, or ACM between groups.

### Occupational/chemical exposures

Five studies have reported on the association between occupational [109] or chemical exposures and NMIBC outcomes [47, 58, 109–111]. None reported statistically significant associations.

## DISCUSSION

In this review, we provided a broad overview of the relationship between various lifestyle factors and NMIBC prognosis. To our knowledge, this is the most comprehensive review of its kind, with over 100 publications on lifestyle factors included.

Among the risk factors discussed, smoking is most convincingly associated with prognosis [112]. Studies suggest that smoking avoidance may reduce recurrence and progression. However, some ambiguity exists regarding cessation, cumulative exposure, and mortality outcomes. This may be related to the “field cancerization” theory, suggesting that long-term carcinogenic insults to the bladder urothelium overshadow the benefit of cessation upon diagnosis [113]. Still, the possibility of harm in bladder cancer prognosis, coupled with the well-established harm to other organ systems, suggests that avoidance and cessation counseling is warranted [63, 114].

Compared to a healthy BMI, being overweight or obese appears to be associated with an increased risk of recurrence and progression. Multiple mechanisms that could explain the associations have been proposed. Elevated BMI could contribute to carcinogenesis through inflammation. Higher BMI is associated with high levels of insulin, insulin-like growth factor (IGF), steroid hormones, and cytokines, all of which can contribute to an increased rate of oncogenic transformation through chronic activation of metabolic signaling cascades [115]. Additionally, an increased neutrophil-to-lymphocyte ratio and elevated C-reactive protein levels, serologic markers of systemic inflammation, are more commonly found in patients with elevated BMI [116]. Finally, high BMI may also make procedures more technically challenging, limiting visualization and risking residual tumor following intravesical procedures [66, 74]. Despite these plausible mechanisms, it has also been proposed that higher BMI, and thus higher energy reserves, may improve rehabilitation following invasive procedures [75]. Regardless, there exists enough evidence to suggest that overweight and obese patients should be counseled on strategies to reduce BMI. Even given the conflicting evidence on bladder cancer prognosis, the known detrimental association between BMI and other health outcomes suggests that achieving a healthy BMI should be encouraged [117, 118]. Given the findings reported in studies on sarcopenia, further investigation of the negative impact of frailty and muscle mass is warranted.

Many studies suggest that DMII is associated with an increased risk of recurrence and progression among patients with NMIBC. Mixed NMIBC/MIBC studies (i.e., patients with more advanced disease) also suggest that DMII could be associated with increased risk of CSM and ACM. The mechanisms that may underlie associations with DMII overlap with those hypothesized for elevated BMI. Patients with DMII also have elevated insulin and IGF-I due to insulin resistance, which subsequently stimulates cell proliferation and inhibits apoptosis [119–121]. Increased circulating IGF-I may be associated with increased bladder cancer risk, although evidence from case-control studies is conflicting [122, 123]. More generally, hyperglycemia may dysregulate energy balance, which affects intracellular metabolism and impairs immune function, leading to increased risk of multiple different cancer types [124]. Metformin may also reduce recurrence and CSM, though no other antidiabetic agents have been shown to improve outcomes. Its use has been connected with a reduced risk of multiple cancer types [125]. Lin et al. reported that, in a large cohort of patients with DMII matched with control participants, metformin use was associated with a dramatically reduced overall cancer risk compared to those without DMII in a dose-dependent fashion [125]. Metformin indirectly reduces mammalian target of rapamycin (mTOR) signaling, which inhibits cancer cell growth and proliferation [126, 127]. Another group suggested that metformin may help prevent stem cell repopulation in bladder cancer, thereby preventing progression [128]. Given findings from basic science research, investigators are exploring potential synergistic effects between metformin and chemotherapeutics like gefitinib to treat multiple cancer types [129].

Statins have been proposed as anti-cancer agents given multiple potential antitumorigenic effects, including angiogenesis suppression, tumor growth inhibition, cell cycle arrest, and apoptosis induction [130–133]. The majority of studies, however, suggest that statins are not associated with bladder cancer-specific outcomes. Still, given their cardiovascular benefit, statins may reduce ACM among patients with bladder cancer [134].

It has been hypothesized that FCIs may reduce the effectiveness of BCG [135]. Studies in murine models have suggested that fibronectin is important for intravesical binding of BCG, meaning that disruption of fibronectin might reduce the ability of BCG to induce the immune response necessary for bladder cancer treatment [135, 136]. Research in humans has not supported the hypothesis. Studies over the past 25 years suggest that FCIs are not associated with bladder cancer outcomes. This understanding is critical given that many patients require drugs like aspirin for cardiovascular health or warfarin to combat cancer-induced hypercoagulability [137, 138].

Regarding dietary exposures, uncooked broccoli has been reported to have prognostic benefit. Broccoli is rich in isothiocyanates which have demonstrated chemopreventive effects in bladder cancer in cell lines and mouse models [139]. The diminished benefit of cooked broccoli is consistent with our understanding of the breakdown of beneficial phytochemicals when exposed to high temperatures [140]. Fluid intake does not appear to be associated with NMIBC outcomes; however, the literature supporting this conclusion is much less robust than the literature reporting a lack of association between fluid intake and bladder cancer risk [141]. The association between alcohol intake and worse NMIBC outcomes is weak, and its effect on other aspects of health is heterogeneous. Some cardiovascular benefit has been observed, but alcohol consumption has also been linked to multiple cancer types [142]. Vitamin E supplementation was beneficial in one study, perhaps through its anti-inflammatory properties [143]. Still, its harmful influence on other cancer types should caution routine supplementation recommendation [144].

Among dietary exposures, L. casei probiotic supplementation had the most promising effect on bladder cancer outcomes, with all three available RCTs reporting reduced risk of recurrence [52, 107, 108]. A significant amount of research is emerging regarding the association of the gastrointestinal and genitourinary microbiome with genitourinary malignancy [145]. A proposed mechanism of benefit that underlies L. casei probiotic supplementation is the improved balance of intestinal flora, which in turn prevents production and promotes elimination of carcinogens [146]. For example, L. casei may prevent urinary excretion of carcinogens that are generated following the consumption of fried ground beef [147]. Additionally, L. casei may enhance immune activity [148]. Future directions include trials among patients undergoing BCG therapy and among patients with MIBC. Additionally, there is a need for well-designed epidemiologic studies investigating the association between bladder cancer outcomes and dietary sources of L. casei (e.g., yogurt, fermented milk, certain cheeses).

Finally, despite the known increased risk of bladder cancer incidence with certain occupational and environmental exposures (e.g., aromatic hydrocarbons, N-nitrosamines), no study reported an association with outcomes among patients with NMIBC [149].

Although a multitude of lifestyle factors have been studied, there are limitations among individual studies, and gaps in our knowledge remain. For example, no studies have evaluated physical activity, which has shown substantial prognostic benefit in other cancer types [150, 151]. Furthermore, those lifestyle factors with retrospective observational evidence of benefit should be confirmed in additional rigorously conducted prospective studies and, if resources are available, RCTs. Evidence from RCTs can be especially useful for revealing true causal associations in factors with previously ambiguous associations. For example, despite prior evidence suggesting a potential benefit, vitamin E was found to increase risk of prostate cancer in the Selenium and Vitamin E Cancer Prevention Trial [144, 152]. Once a final consensus on lifestyle recommendations in bladder cancer is reached, implementation strategies should be investigated to help patients make long-lasting behavior changes. Lifestyle studies of other cancer types have begun to explore health monitoring technology and online behavior change resources for this purpose [153, 154]. Additionally, deficits in patient knowledge of the association between lifestyle and bladder cancer warrants improved methods of patient education. For example, Neider et al. prospectively surveyed patients presenting for urologic evaluation on their knowledge of risk factors for various cancers [155]. Only 36% reported smoking as a risk factor for bladder cancer compared to 98% reporting smoking as a risk factor for lung cancer. Patients were less likely to report smoking as a risk factor for bladder cancer if they were not college educated, were older (60–80 years old), or were male. Furthermore, Winters et al. reported that, among patients with bladder cancer in the Surveillance, Epidemiology, and End Results (SEER) database, there was no significant difference in the adjusted odds ratio of smoking cessation among patients with bladder cancer compared to noncancer controls [156]. This further demonstrates a need for the dissemination of educational resources for patients with bladder cancer as well as implementation strategies for lifestyle modifications. Finally, it is important to investigate the effect of lifestyle interventions on patient quality of life and functional status and, in turn, how improvements in these important patient-reported outcomes might affect prognosis.

A limitation of this review that warrants acknowledgment is the large number of studies identified via review of publication citations relative to those studies identified in our search. Regardless, the scope of this review is quite comprehensive and includes more studies on individual factors than many prior reviews. Still, it is possible that studies on some individual factors may not have been identified. Additionally, we did not search databases other than PubMed, and thus studies of individual factors not available in this database may have been overlooked.

## CONCLUSION

Available literature on the associations between lifestyle factors and bladder cancer outcomes suggests that smoking avoidance/cessation, healthy BMI, DMII prevention and treatment, and L. casei supplementation may be beneficial. The use of most diabetic drugs, FCIs, and statins appears to be safe and does not affect treatment effectiveness. Further research is needed to support possible benefits of uncooked broccoli consumption; metformin use; supplemental vitamin E use; and possible harms from diets high in fried foods and red meat, supplemental vitamin B9 use, areca nut chewing, and glyburide use. Evidence did not otherwise suggest associations for other dietary items (individual or multimodal), beverages, or nutritional supplements; fluid intake; or occupational or chemical exposures.

## Supplementary Material

Supplementary Table 1

## Figures and Tables

**Table 1:** Factors with evidence beneficial associations with NMIBC outcomes

Factor	Evidence summary
Healthy BMI***	Multiple studies have reported elevated BMI (≥25 kg/m^2^) is associated with increased risks of recurrence and progression, with more ambiguous results suggesting increased risks of CSM and ACM. Two studies reported that sarcopenia may be a better body composition metric for predicting outcomes than BMI.
*Lactobacillus casei* supplementation***	Three RCTs reported supplementation reduces risk of recurrence.
Smoking avoidance/cessation***	Multiple studies have reported that current smoking is associated with recurrence. Current and former smoking may be associated with CSM. Long-term smoking cessation may be associated with reduced recurrence and progression.
DMII prevention and treatment**	Associations are ambiguous; however, a few studies have reported associations with improved outcomes among patients with DMII treated with metformin.
Broccoli (uncooked)**	One study reported higher intake was associated with reduced CSM and ACM.
Supplemental vitamin B9 (folate) avoidance**	One study reported supplementation was associated with an increased risk of recurrence.
Supplemental vitamin E**	One study reported supplementation was associated with a reduced risk of recurrence.
“Western diet” avoidance**	One study reported a diet high in red meat and fried foods was associated with an increased risk of recurrence.
Areca nut chewing avoidance*	One study reported that heavy use (>10 nuts/day) was associated with increased risk of recurrence.
Glyburide avoidance*	One study reported use was associated with increased risk of CSM.

ACM= All-cause mortality, BMI = Body mass index, CSM = Cancer-specific mortality, DMII = Diabetes Mellitus type II, NMIBC = Nonmuscle invasive bladder cancer, RCT = Randomized controlled trial.

* Evidence from retrospective studies suggests benefit.

** Evidence from prospective studies or non-randomized trial suggests benefit.

*** Evidence from meta-analyses and/or randomized controlled trials suggests benefit.
