# Supplementary material for: Lifestyle and Non-muscle Invasive Bladder Cancer Recurrence, Progression, and Mortality: Available Research and Future Directions
Source: Bladder Cancer. Author manuscript; Available in PMC 2021 Jun 3. (PMC8174672; doi:10.3233/blc-190249)
Supplement: Supplementary Table 1 [file NIHMS1583231-supplement-Supplementary_Table_1.docx]

Supplementary Table 1: Description of included studies ordered alphabetically by first author.

| **Author and year** | **Study design*** | **Population** | **Definition of primary outcomes*** | **Time period** | **Age in years** | **Follow-up** | **Primary results**** |
| --- | --- | --- | --- | --- | --- | --- | --- |
| Ahirwar et al. - 2008^[1]^ | Case-control | 136 patients with NMIBC/MIBC | Recurrence: Newly found bladder tumor following a previous negative follow-up cystoscopy. | 2004-2007 | Mean/median NR | 13 months (median) | **Smoking**  *Ref. = Never*  *Former*  Recurrence: NS on univariable analysis  *Current*  Recurrence: NS on univariable analysis |
| Ahn et al. - 2016^[2]^ | Retrospective cohort | 645 patients with NMIBC | Recurrence: First tumor recurrence (regardless of grade or stage).  Progression: Any increase in grade (G1/2 to G3) or stage (Ta to T1 or T2, T1 to T2) after repeat transurethral resection for recurrence. | 2004-2015 | 64.6 (median) | 46 months (median) | **BMI**  *Ref. = <25*  Recurrence: NS on univariable analysis  Progression: NS on univariable analysis  **DMII**  *Ref. = No DMII*  Recurrence: OR 1.22 (0.89-1.30)  Progression: OR 1.54 (0.95-2.50) |
| Ajili et al. - 2013^[3]^ | Retrospective cohort | 81 patients with NMIBC | Recurrence: Reappearance of tumor after the initial treatment with at least one tumor-free cystoscopy interval. | 2000-2005 | 60 (median) | 30 months (max.) | **Smoking**  *Ref. = ≤60 pack-years*  *>60 pack-years*  Recurrence: 0.264 (0.110-0.631) |
| Alfthan et al. - 1983^[4]^ | Randomized controlled trial | 30 patients with NMIBC | Prevention: No tumors during the last 12 months of treatment or during the whole treatment period if it was less. Partial prevention was described as disappearance of ≥50% of tumors.  Progression: Increase in number and/or grade of tumors. | NR | 64.1-68.6 (mean) | 17.6 months (mean) | **Etretinate**  *Ref. = Placebo*  Prevention/partial prevention: 11 intervention participants versus 4 control participants (p<0.01)  Progression: 0 intervention participants versus 2 control participants (p<0.01) |
| Allard et al. - 1995^[5]^ | Prospective cohort | 368 patients with NMIBC | Recurrence: Tumor detected on cystoscopy. | 1990-1992 | 65.1 (mean) | 23.7 (mean) | **Smoking**  *Ref. = Never*  *Former*  Recurrence: NS on univariable analysis  *Current*  Recurrence: NS on univariable analysis |
| Aso et al. - 1992^[6]^ | Randomized controlled trial | 48 patients with NMIBC | Recurrence: ND | 1988-1989 | Mean/median NR | 427-428 days (mean) | ***Lactobacillus casei* probiotic**  *Ref. = No use or placebo*  Recurrence at 12 months: 57% (intervention) versus 83% (control) (p<0.01) |
| Aso et al. - 1995^[7]^ | Randomized controlled trial | 125 patients with NMIBC | Recurrence: Cytologic examination, cystoscopy, and/or biopsy.  Progression: Upgrading or upstaging of tumors. | 1990-1991 | Mean/median NR | NR | ***Lactobacillus casei* probiotic**  *Ref. = Placebo*  Recurrence: 2.58 (p=0.013) (HR of being recurrence free)  Progression: 1 intervention participant versus 7 control participants (p<0.01) |
| Bachir et al. – 2014^[8]^ | Retrospective cohort | 847 patients with NMIBC/MIBC | Recurrence: ND  CSM: ND  ACM: ND | 1998-2008 | 63.6-66.3 (mean) | 39 months (mean), 23.4 months (median) | **BMI**  *Continuous*  Recurrence: 0.978 (0.955-1.003)  CSM: 0.989 (0.960-1.019)  ACM: 0.984 (0.958-1.010) |
| Berglund et al. - 2008^[9]^ | Retrospective cohort | 952 patients with NMIBC/MIBC | Recurrence: Visual and/or biopsy proven evidence of recurrence at cystoscopy or a positive repeat cytology.  Progression: Progression to surgery. | 1978-2006 | 65-69 (mean) | 4.3 years (mean of those without recurren-ce) | **Statins**  *Ref. = No use*  Recurrence: 1.04 (0.81-1.34)  Progression: 0.77 (0.52-1.13) |
| Boorjian et al. - 2009^[10]^ | Retrospective cohort | 907 patients with NMIBC/MIBC | Recurrence: Visual and/or biopsy proven evidence of tumor at cystoscopy, or by positive urine cytology.  Progression:  Progression to surgery. | 1990-2006 | 65-71 (median) | 4.2 years (mean of those without recurrence) | **FCI**  *Ref. = No use*  *Any FCI (aspirin, clopidogrel, warfarin)*  Recurrence: 1.01 (0.85-1.19)  Progression: 0.91 (0.71-1.18)  *Aspirin*  Recurrence: 0.91 (0.75-1.10)  Progression: 0.71 (0.52-0.96)  *Clopidogrel*  Recurrence: 1.35 (0.94-1.94)  Progression: 0.70 (0.36-1.35)  *Warfarin*  Recurrence: 1.19 (0.89-1.59)  Progression: 1.89 (1.31-2.74) |
| Bostrom et al. - 2009^[11]^ | Retrospective cohort | 248 patients with NMIBC/MIBC | CSM/ACM: ND | 1986-2005 | 64 (median) | 75 months (mean) | **Smoking**  *Ref. = Non-smoker*  CSM: NS on univariable analysis  ACM: MVNA |
| Byar et al. - 1977^[12]^ | Randomized controlled trial | 118 patients with NMIBC | Recurrence: A visit at which one or more tumors have reappeared in the bladder after having been removed previously by transurethral resection.  Progression:  Increase in number of tumors. Increase in tumor grade. | 1971-1976 | NR | 31 months (mean) | **Pyridoxine**  *Ref. = Placebo*  Recurrence: NS on univariable analysis.  Progression: 22% (intervention) versus 46% (control) had an increase in number of tumors (p=0.026) |
| Cao et al. - 2016^[13]^ | Retrospective cohort | 242 patients with NMIBC | Recurrence: Tumor recurrence after transurethral resection, with or without pathological upstaging or upgrading. | 2008-2013 | 64.2 (mean) | 21 months (mean) | **Areca nut chewing**  *Ref. = None*  *Light (<10/day)*  Recurrence: NS on univariable analysis  *Heavy (>10/day)*  Recurrence: 2.18 (1.37-3.47)  **Smoking**  *Ref. = Never*  *Former*  Recurrence: NS on univariable analysis  *Current* Recurrence: 3.09 (1.99-4.80)  **DMII**  *Ref. = No DMII*  *DMII*  Recurrence: NS on univariable analysis |
| Carpenter et al. - 1989^[14]^ | ND | 100 patients with NMIBC/MIBC | Recurrence: ND  Progression: Progression to cystectomy or partial cystectomy  CSM/ACM: ND | 1982-1986 | 64 (years) | 4.3 years (mean) | **Smoking**  *Ref. = Non-smoker*  Recurrence: MVNA  Progression: NS on univariable analysis  CSM/ACM: NS on univariable analysis |
| Carta et al. - 2018^[15]^ | Retrospective cohort | 160 patients with NMIBC | Recurrence: Histological confirmation of a newly found bladder or prostatic urethra tumor following at least one tumor-negative follow-up cystoscopy or two surgical resection sessions for the primary tumor.  Progression: Transition from low- to high-grade, increase in TNM staging, progression to cystectomy, or “uncontrollable” disease. | 1997-2000 | Mean/median NR | 4.63 years (mean) | **Aromatic amines**  *Ref. = No exposure*  *Exposure*  Recurrence: 1.129 (0.743-1.743)  Progression: 0.719 (0.382-1.351)  **PAH**  *Ref. = No exposure*  *Exposure*  Recurrence: 1.077 (0.701-1.654)  Progression: 0.690 (0.358-1.327) |
| Chade et al. - 2010^[16]^ | Retrospective cohort | 155 patients with NMIBC | Recurrence: ND  Progression: Progression to invasive bladder cancer, defined as cT1 or higher (≥cT1) and progression to MIBC, defined as cT2 or higher (≥cT2), or radical cystectomy. | 1990-2008 | 69 (median) | 3.3-4.0 years (median) | **Smoking**  *Ref. = Never*  *Former*  Recurrence: NS on univariable analysis  Progression: NS on univariable analysis  *Current*  Recurrence: NS on univariable analysis  Progression: NS on univariable analysis |
| Chen et al. - 2007^[17]^ | NR | 265 patients with NMIBC | Recurrence: Histologically confirmed recurrent bladder cancer detected >8 weeks after the initial TUR.  Progression:  Recurrent cancer that invaded into the muscle layer. | 1997-2005 | 65-69 (median) | 38 months (median) | **Smoking - categorical**  *Ref. = Quitters (quit within 1 year before and 3 months after diagnosis)*  *Never*  Recurrence:2.2 (1.1-4.5)  Progression: NS on univariable analysis  *Former*  Recurrence: 1.4 (0.7-2.7)  Progression: NS on univariable analysis  *Current*  Recurrence: 2.2 (1.2-4.0)  Progression: MVNA  **Smoking – cumulative**  *Ref. = 1-19 pack-years*  *20-39 pack-years*  Recurrence: NS on univariable analysis  Progression: NS on univariable analysis    *40-59 pack-years*  Recurrence: NS on univariable analysis  Progression: NS on univariable analysis  ≥*60 pack-years*  Recurrence: MVNA  Progression: MVNA |
| Cheng et al. - 1999^[18]^ | Retrospective cohort | 83 patients with NMIBC | Progression: The development of muscle-invasive or more advanced stage carcinoma, distant metastasis, or death from bladder cancer.  CSM: Included in definition of progression. | 1987-1992 | 71 (mean) | 5.2 years (mean) | **Alcohol**  Progression: Never, former, and current NS different on univariable analysis.  CSM: Never, former, and current NS different on univariable analysis.  **Smoking*****  *Ref. = Non-smoker*  Progression: Never, former, and current NS different on univariable analysis.  CSM: Never, former, and current NS different on univariable analysis. |
| Chromecki et al. - 2013^[19]^ | Retrospective cohort | 4,118 patients with NMIBC/MIBC | Recurrence: ND  CSM: ND  ACM: ND | 1979-2008 | 67 (median) | 44 months (median) | **BMI**  *Ref. = <25*  *25.9-29.9*  Recurrence: 0.91 (0.76-1.06)  CSM: 0.80 (0.68-0.95)  ACM: 1.40 (1.23-1.57)  *>30*  Recurrence: 1.67 (1.46-1.91)  CSM: 1.43 (1.24-1.66)  ACM: 1.81 (1.60-2.05) |
| Crivelli et al. - 2013^[20]^ | Retrospective cohort | 1,117 patients with NIMBC | Recurrence: Defined as the first tumor relapse in the bladder regardless of stage.  Progression: A muscle-invasive relapse in the bladder.  CSM/ACM: Cause of death was determined by the treating physicians, chart review corroborated by death certificates or death certificates alone. | 1996-2007 | 65 (mean), 67 (median) | 62.7 months (median) | **Statins**  *Ref. = No use*  Recurrence: NS on univariable analysis  Progression: NS on univariable analysis  CSM: NS on univariable analysis  ACM: NS on univariable analysis |
| da Silva et al. - 2013^[21]^ | Retrospective cohort | 1,502 patients with NMIBC/MIBC | Recurrence: Tumor relapse in the operative field, regional lymph nodes and/or distant metastasis.  CSM: Cause of death was determined by treating physicians by chart review corroborated by death certificates or by death certificates alone. | 1992-2008 | 65.5 (mean), 66 (median) | 34 months (median) | **BMI**  *Continuous*  Recurrence: 1.05 (1.03-1.07)  CSM: 1.05 (1.02-1.07)  **Smoking**  *Ref. = Never*  *Former*  Recurrence: 1.27 (0.98-1.65)  CSM: 1.24 (0.98-1.66)  *Current*  Recurrence: 1.47 (1.12-1.92)  CSM: 1.43 (1.06-1.93)  **Statins**  *Ref. = No use*  Recurrence: 1.04 (0.86-1.24)  CSM: 1.04 (0.84-1.28) |
| Dabi et al. - 2017^[22]^ | Retrospective cohort | 701 patients with NMIBC/MIBC | Recurrence: Tumor relapse in the operative field, regional lymph nodes and/ or distant metastases.  CSM: Death cause was determined using patient’s death certificate. | 1995-2011 | 65.2 – 66.8 (mean) | 45 months (median) | **BMI**  *Ref. = 18-25*  *>25 - 30*  Recurrence: 1.14 (0.78–1.66)  CSM: 1.13 (0.74–1.74)  *>30*  Recurrence: 1.58 (1.06-2.34)  CSM: 1.58 (1.01–2.48) |
| Decensi et al. - 2000^[23]^ | Randomized controlled trial | 99 patients with NMIBC | Recurrence: Presence of a papillary tumor or an infiltrating cancer at the time of cystoscopy. | 1993-1994 | 61.6-63.8 (mean) | NR | **Fenretinide**  *Ref. = Placebo*  Recurrence: 27 participants in the intervention group versus 21 participants in the control group (p=0.36) |
| Donat et al. - 2003^[24]^ | Prospective cohort | 267 patients with NMIBC | Recurrence: ND | 1998-2001 | 69.1 (median) | 2.6 years (median) | **Fluid intake**  *Continuous*  Recurrence: NS on univariable analysis  **Smoking**  *Ref. = Never*  *Former*  Recurrence: 0.94 (0.65-1.35)  *Current*  Recurrence: 1.10 (0.67-1.82) |
| Ferro et al. - 2018^[25]^ | Retrospective cohort | 1,115 patients with NMBIC | Recurrence: The appearance of any tumor.  Progression: Muscle-invasive disease during follow up.  CSM: ND  ACM: ND | 2002-2012 | 71 (median) | 26 months (median) | **BMI**  *Ref. = 18.5-<25*  *<18.5*  Recurrence: 0.27 (0.06-1.11)  Progression: 0.64 (0.15-2.66)  CSM: NS  ACM: NS  *25-29.99*  Recurrence: 4.00 (3.18-5.01)  Progression: 2.52 (1.85-3.42)  CSM: NS  ACM: NS  ≥*30*  Recurrence: 5.33 (4.16-6.83)  Progression: 2.51 (1.76-3.57)  CSM: NS  ACM: NS |
| Fleshner et al. - 1999^[26]^ | Retrospective cohort | 286 patients with NMIBC | Recurrence: A recurrent lesion in the bladder at least 3 months after the initial transurethral resection.  Progression: The development of MIBC, the development of metastases, or the development of uncontrollable NMIBC that was unamenable to conservative therapy. | 1985-1995 | 58.7-63.7 (mean) | 55.2-59.1 months (mean) | **Smoking**  *Ref. = Former*  *Quitter (pa tients with 20 pack-years of exposure and who quit smoking between 1 year prior to and up to 3 months following the diagnosis)*  Recurrence: 0.99 (0.77-1.25)  Progression: NS on univariable analysis  *Current*  Recurrence: 1.40 (1.03-1.91)  Progression: MVNA |
| Gee et al. – 2008^[27]^ | Retrospective cohort | 43 patients with NMIBC | Recurrence: First tumor recurrence.  Progression: Progression in stage to lamina propria invasion or more advanced stages. | 1991-2003 | 63-72 (mean) | NR | **Aspirin**  *Ref. = No use*  Recurrence: 0.179 (0.062-0.516)  Progression: NS on univariable analysis  **Smoking**  *Ref. = Never*  *Former*  Recurrence: 3.199 (0.981-10.433)  *Current*  Recurrence: 0.270 (0.082-0.889) |
| Gierth et al. - 2018^[28]^ | Prospective cohort | 678 patients with NMIBC/MIBC | CSM/ACM: The cause of death was determined by the treating physician, by chart review corroborated by death certificates, or by death certificates alone. | 2011 | 70 (median) | At least 21 months | **BMI**  *Ref. = <25*  *25-29.9*  CSM: 1.12 (0.73-1.71)  ACM: 0.80 (0.56-1.13)  *>30*  CSM: 0.71 (0.42-1.19)  ACM: 0.60 (0.39-0.92) |
| Goossens et al. - 2016^[29]^ | Randomized controlled trial | 292 patients with NMIBC | Recurrence: The new occurrence of tumor at the same or at a different site as the index cancer.  Progression: Recurrence with an increase in tumor grade, or an increase in TNM stage, or a new occurrence of carcinoma in situ in the bladder previously free from such lesions, or a new occurrence of multiple tumors following resection of a solitary tumor, or the need for a cystectomy because of refractory disease. | 2009-2013 | 68 (median) | 17.93 months (median) | **Selenium**  *Ref. = Placebo*  Recurrence: 0.85 (0.56-1.29)  Progression: 1.48 (0.65-3.38) |
| Grotenhuis et al. - 2014^[30]^ | Retrospective cohort | 963 patients with NMIBC | Recurrence: New, histologically confirmed bladder or prostatic urethra tumor following at least 1 tumor-negative follow-up cystoscopy result or 2 surgical resection sessions for the primary tumor.  Progression: First occurrence of grade progression, stage progression, occurrence of local metastasis or distant metastasis or both, and  cystectomy for therapy-resistant disease. | 1995-2010 | 61-66 (mean) | 3.7 years (median) | **Smoking**  *Ref. = Never*  *Ever*  Recurrence: 1.06 (0.80-1.41)  Progression: 1.15 (0.72-1.84)  *Former*  Recurrence: 1.14 (0.85-1.53)  Progression: 1.36 (0.84-2.21)  *Current*  Recurrence: 0.93 (0.67-1.29)  Progression: 0.80 (0.45-1.42) |
| Hoffman et al. - 2006^[31]^ | Retrospective cohort | 84 patients with NMIBC | Recurrence: Number of total recurrences.  Progression: Tumor progression, the time to cystectomy, and the time to the development of distant metastases. | NR | 65 (median) | 46 months (median) | **Statins**  *Ref. = No use*  Recurrence: NS on UV analysis  Progression: MVNA |
| Holz et al. - 2017^[32]^ | Retrospective cohort | 123 patients with NMIBC | Recurrence: Reappearance of tumor (any grade and any stage) during follow-up.  Progression: Progression to MIBC, development of lymph node (N+) disease or distant metastasis (M1). | 1998-2012 | 68 (mean), 69 (median) | 49 months (median) | **Smoking**  *Ref. = Never*  *Former*  Recurrence: MVNA  Progression: NS on univariable analysis  *Current*  Recurrence: MVNA  Progression: NS on univariable analysis |
| Hou et al. - 2017^[33]^ | Meta-analysis | 10,192 patients with NMIBC/MIBC | Recurrence: ND  Progression: ND  CSM: ND | 1995-2015 | 61.6-75.0 (mean) | 23.7-80.9 months (mean) | **Smoking**  *Ref. = Never*  *Former*  Recurrence: SSRE 1.22 (1.09-1.37)  Progression: SSRE 1.16 (0.92-1.46)  CSM: SSRE 1.20 (1.03-1.41)  *Current*  Recurrence: SSRE 1.23 (1.05-1.45)  Progression: SSRE 1.11 (0.70-1.75)  CSM: SSRE 1.28 (1.07-1.52) |
| Hudson et al. – 1990^[34]^ | Retrospective cohort | 149 patients with NMIBC | Recurrence: Recurrence following BCG  Progression: Invasive or metastatic disease. | 1981-1989 | NR | 29.8 months (median) | **FCIs (Aspirin, aspirin plus dipyramidole, indomethacin, ibuprofen, warfarin)**  *Ref. = No use*  Recurrence: MVNA  Progression: NS on univariable analysis |
| Hwang et al. - 2011^[35]^ | Retrospective cohort | 251 patients with NMIBC | Recurrence: First tumor recurrence (regardless of grade or stage).  Progression: Any increase in grade (G1/2 to G3) or stage (Ta to T1 or T2, T1 to T2). | 2000-2010 | 67 (median) | 34 months (median) | **Smoking**  *Ref. = Non-smoker*  Recurrence: 1.63 (1.1-2.5)  Progression: NS on univariable analysis  **DMII**  *Ref. = No DMII*  Recurrence: 2.11 (1.4-3.2)  Progression: 9.35 (3.1-28.6) |
| Jochems et al. - 2018^[36]^ | Prospective cohort | 716 patients with NMIBC | Recurrence: The new occurrence of NMIBC (stage Ta, T1, or pTis) at the same or at a different site as the initial primary bladder tumor and excluding recurrence identified at the first check cystoscopy. | 2005-2011 | 71 (median) | 3.7 years (mean) | **Alcohol**  *Ref. = No use*  *25-125mL/day*  Recurrence: 0.90 (0.63-1.27)  *>125mL/day*  Recurrence: 0.97 (0.70-1.36)  **Fluid intake**  *Ref. = 250-850mL/day*  *850-1200mL/day*  Recurrence: 1.17 (0.85-1.62)  *>1200mL/day*  Recurrence: 0.98 (0.70-1.38) |
| Jochems et al. - 2018^[37]^ | Prospective cohort | 728 patients with NMIBC | Recurrence: The new occurrence of a NMIBC (stage Ta, T1, or pTis) at the same or at a different site as the initial pri- mary bladder tumor and excluding recurrence identified at the first check cystoscopy. | 2005-2011 | 69 (mean) | 3.7 years (median) | **Fruits and vegetables**  *Ref. = Tertile 1*  *Tertile 2*  Recurrence: 1.09 (0.79-1.50)  *Tertile 3* Recurrence: 1.07 (0.78-1.47)  **Total fruits**  *Ref. = Tertile 1*  *Tertile 2*  Recurrence: 1.22 (0.89-1.69)  *Tertile 3* Recurrence: 0.85 (0.63-1.14)  **Total vegetables**  *Ref. = Tertile 1*  *Tertile 2*  Recurrence: 0.97 (0.70-1.33)  *Tertile 3* Recurrence: 1.02 (0.74-1.41) |
| Kamat et al. - 2007^[38]^ | ND | 156 patients with NMIBC | Recurrence: ND  Progression: ND  ACM: ND | NR | NR | 56 months (median) | **Statins**  *Ref. = No use*  Recurrence: NS on univariable analysis  Progression: NS on univariable analysis  ACM: NS on univariable analysis |
| Kashif Khan et al. - 2014^[39]^ | Retrospective cohort | 64 patients with NMIBC | Recurrence: Tumors of the same initial stage at cystoscopy.  Progression: Tumor involved the detrusor muscle, had nodal or distant metastasis. | 2008-2012 | 59.86 (mean) | 28.36 months (mean) | **Smoking**  *Ref. = Non-smoker*  Recurrence: NS on univariable analysis  Progression: OR 4.02 (1.01-15.88) |
| Kelly et al. - 2019^[40]^ | Randomized controlled trial | 427 patients with NMIBC | Recurrence: Confirmation of cancer recurrence by cystoscopy. | 2007-2012 | 67 (median) | 44 months (median) | **Celecoxib**  *Ref. = Placebo*  Recurrence: 0.82 (0.60-1.12)  Progression: 10% versus 9.7% (log-rank p=0.8)  ACM: 1.21 (0.68-2.15) |
| Kluth et al. - 2013^[41]^ | Retrospective cohort | 892 patients with NMIBC | Recurrence: First tumor relapse in bladder or prostatic urethra regardless of tumor stage.  Progression: Tumor relapse at tumor stage T2 or higher in the bladder or prostate.  CSM/ACM: The cause of death was determined by treating physicians, by chart review corroborated by death certificates or by death certificates alone. | 1996-2007 | 68 (median) | 42.8 months (median) | **BMI**  *Continuous*  Recurrence: 1.07 (1.04-1.09)  Progression: 1.08 (1.04-1.12)  CSM: 1.29 (1.20-1.37)  ACM: 1.06 (1.04-1.09)  *Ref. = <30*  ≥*30*  Recurrence: 2.66 (2.12-3.32)  Progression: 1.49 (1.00-2.21)  CSM: 3.15 (1.74-5.67)  ACM: 1.42 (1.06-1.92) |
| Koch et al. - 1986^[42]^ | Prospective cohort | 761 patients with NMIBC | Recurrence: Histologically-proven diagnosis. | 1977-1983 | Mean/median NR | 59 months (median) | **Artificial sweeteners**  *Ref. = No use*  Recurrence: NS (hazard ratio NA)  **Chemical exposure**  *Ref. = No exposure*  Recurrence: NS (hazard ratio NR)  **Coffee**  *Ref. = No use*  Recurrence: NS (hazard ratio NR)  **Smoking**  *Ref. = Non-smoker*  Recurrence: NS (hazard ratio NR) |
| Korkes et al. - 2010^[43]^ | Retrospective cohort | 99 patients with NMIBC | Recurrence: ND  Progression: Progression to MIBC  CSM: ND | 1994-2000 | 67 (mean) | 49.3 months (median) | **Smoking - categorical**  *Ref. = Never*  *Current*  Progression: NS on univariable analysis  NS on Chi-square between former smokers, Early-quitters (quit *≤*1 year after diagnosis), Late-quitters (quit >1 year after diagnosis), and current smokers  ***Smoking – cumulative***  *Ref. = <60 pack years*  *>60 pack years*  Progression: MVNA  CSM: NS on univariable analysis |
| Koshiaris et al. - 2017^[44]^ | Retrospective cohort | 1,733 patients with bladder cancer (stage unspecified) | CSM/ACM: Clinical Practice Research Datalink record and also from the UK national system of recording death provided by the Office for National Statistics. | 1999-2013 | 65.39-67.49 (mean) | NR | **Smoking**  *Ref. = Quitters*  *Current*  CSM: 1.14 (0.71-1.83)  ACM: 0.86 (0.44-1.65) |
| Lacombe et al. - 2016^[45]^ | Retrospective cohort | 189 patients with NMIBC | Recurrence: a pathologically confirmed (at re-TURBT) new tumor(s) identified during cystoscopy follow-up after TURBT. | 1990-1992, 1997-2002 | 62.8 (mean) | 5.6 years (mean) | **Smoking**  *Ref. = Never*  *Former*  Recurrence:  2.76 (1.03-7.40)  *Current*  Recurrence: 2.93 (1.08-7.94) |
| Lamm et al. - 1994^[46]^ | Randomized controlled trial | 65 patients with bladder cancer (stage unspecified) | Recurrence: Presence of recurrent tumors resected transurethrally and confirmed by microscopic examination.  ACM: ND | 1985-1992 | 65.9-68.1 (mean) | 45 months (mean) | **Megadose multivitamins**  *Ref. = Recommended daily allowance*  Recurrence (5-year estimates): 80% (intervention) versus 40% (control) (p=0.0014)  ACM (survival rate): 76% (intervention) versus 74% (control) (NS) |
| Lammers et al. - 2011^[47]^ | Prospective cohort | 718 patients with NMIBC | Recurrence: Recurrence during treatment period, recurrence after treatment period, and occurrence of CIS. | 1998-2004 | 66.2-66.6 (mean) | 2.5 years (mean) | **Smoking**  *Ref. = Never*  *Current/former*  Recurrence (European Organization for Research and Treatment of Cancer risk factors included in multivariable analysis): 1.47 (1.00-2.15)  Recurrence (Club Urologico Espanol de Tratamiento Oncologico risk factors included in multivariable analysis): 1.57 (1.06-2.31) |
| Lee et al. - 2011^[48]^**** | Retrospective cohort | 602 patients with NMIBC/MIBC | Recurrence/progression: Local recurrence at or below the common iliac bifurcation or distant metastasis documented by imaging and biopsy, if indicated. | 1989-2008 | 60.5-64.3 (mean) | 56 months (median) | **Smoking - binary**  *Ref. = Non-smoker*  Recurrence/progression: 0.94 (p=0.697)  CSS: 1.10 (p=0.587)  ACM: 1.01 (p=0.930)  **Smoking – categorical**  *Ref. = Never*  *Former*  Recurrence/progression: 0.93 (0.66-1.29)  CSS: 1.21 (0.86-1.70)  *Current*  Recurrence/progression: 0.91 (0.63-1.31)  CSS: 0.94 (0.64-1.37)  **Smoking – cumulative**  *Ref. = Never*  *<10 pack-years*  Recurrence/progression: 1.55 (0.74-3.27)  CSS: 1.38 (0.58-3.28)  *<20 pack-years*  Recurrence/progression: 0.59 (0.30-1.16)  CSS: 0.68 (0.34-1.39)  *<30 pack-years*  Recurrence/progression: 0.91 (0.50-1.65)  CSS: 1.02 (0.56-1.85)  *<40 pack-years*  Recurrence/progression: 0.98 (0.55-1.74)  CSS: 1.15 (0.65-2.02)  ≥*40 pack-years*  Recurrence/progression: 0.86 (0.56-1.31)  CSS: 0.95 (0.61-1.48) |
| Leibovici et al. – 2015^[49]^ | Case-control | 519 patients with NMIBC and 505 healthy control participants | Recurrence: Newly found bladder tumor following a previous negative follow-up cystoscopy.  Progression: The transition from NMIBC to MIBC or metastatic disease. | 1995-2003 | 62.3-65.7 (mean) | 20.8 months (median) | **Smoking - categorical**  *Ref. = Never*  *Former*  Recurrence: 1.11 (0.73-1.70)  Progression: 1.30 (0.53-3.16)  *Current/recent quitter*  Recurrence: 0.81 (0.47-1.37)  Progression: 0.59 (0.17-2.03)  **Smoking – cumulative**  Recurrence: 1.00 (0.99-1.00)  Progression: 1.01 (1.00-1.02) |
| Lenis et al. – 2018^[50]^ | Retrospective cohort | 90 patients with NMIBC | Recurrence: The presence of urothelial carcinoma on biopsy or repeat resection.  Progression: Any increase in grade or stage of disease. | 2012-2015 | 69.7 (mean) | 20 months (median) | **BMI**  *Ref. = <30*  ≥*30*  Recurrence or progression: 3.42 (1.55-7.52)  **DMII**  *Ref. = No DMII*  Recurrence or progression: 1.09 (0.46–2.59) |
| Li et al. - 2017^[51]^ | Retrospective cohort | 484 patients with NMIBC | Recurrence: Histologically-confirmed tumor recurrence.  Progression: Pathological stage ≥T2 or disease metastasis. | 2007-2015 | 64 (median) | 25 months (median) | **Smoking - categorical**  *Ref. = Never*  *Former*  Recurrence: 0.970 (0.639-1.471)  Progression: NS on univariable analysis  *Current*  Recurrence: 1.487 (0.948-2.331)  Progression: NS on univariable analysis  **Smoking – categorical**  *Ref. = Current*  *Quit <10 years prior to diagnosis*  Recurrence: 0.937 (0.620-1.415)  *Quit* ≥*10 years prior to diagnosis*  Recurrence: 0.456 (0.257-0.809)  **Smoking – cumulative**  *Ref. = cigarette index <400*  *Cigarette index* ≥*400*  Recurrence: 2.409 (1.487-3.903) |
| Lipsky et al. - 2013^[52]^ | Retrospective cohort | 224 patients with NMIBC | Recurrence: Biopsy-proven recurrence within the bladder.  Progression: Stage increase at the time of recurrence.  CSM/ACM: ND | 2001-2011 | 68-78 (mean) | 44.1 months (median) | **Aspirin**  *Ref. = No use*  Recurrence: NS on univariable analysis  Progression: NS on univariable analysis  CSM/ACM: NS on univariable analysis  **Clopidogrel**  *Ref. = No use*  Recurrence: NS on univariable analysis  Progression: NS on univariable analysis  CSM/ACM: NS on univariable analysis  **Warfarin**  *Ref. = No use*  Recurrence: NS on univariable analysis  Progression: NS on univariable analysis  CSM/ACM: NS on univariable analysis |
| Lopez-Beltran et al. – 1992^[53]^ | Retrospective cohort | 36 patients with NMIBC/MIBC | CSM: ND | NR | 64.4 (mean) | 56.75 months (mean) | **Smoking**  *Ref. = Non-smoker*  CSM: NS on univariable analysis |
| Lukas et al. - 2017^[54]^**** | Case-control | 143 patients with NMIBC/MIBC and 337 controls | Recurrence: ND | NR | 60.36-70.4 (median), 59.94-69.24 (mean) | NR | **Aromatic amine**  *Ref. = Not exposed*  *Exposed*  Recurrence: 1.58 (0.66-3.74)  **Azo dyes**  *Ref. = Not exposed*  *Exposed*  Recurrence: 0.92 (0.51-1.65)  **PAH**  *Ref. = Not exposed*  *Exposed*  Recurrence: 0.75 (0.42-1.33) |
| Maurer et al. - 2009^[55]^ | Retrospective cohort | 390 patients with NMIBC/MIBC | ACM: ND | 1986-2004 | 68 (median) | NR | **BMI**  *Ref. = Normal weight*  ACM: MVNA |
| Mazdak et al. - 2012^[56]^ | Randomized controlled trial | 46 patients with NMIBC | Recurrence: ND | 2006-2010 | 59.16-60.62 (mean) | 17.8-24.57 (mean) | **Vitamin E**  *Ref. = No use*  Recurrence: RR 0.53 (0.19-0.92) |
| Michalek et al. - 1985^[57]^ | NR | 302 patients with NMIBC/MIBC | Recurrence: Determined from patient’s medical record.  ACM: Review of medical records. | 1063-1975 | 66 (mean) | NR | **Smoking**  *Ref. = Never*  *Former/Current*  Recurrence: NS on univariable analysis  ACM: Regression coefficient=0.012, SE=0.092, p=0.89 |
| Michalek et al. - 1987^[58]^ | Prospective cohort | 102 patients with NMIBC | Recurrence: ND | 1960-1965 | NR | NR | **Smoking**  *Ref. = Non-smoker*  Recurrence: NS on univariable analysis  **Vitamin A**  *Ref. = Low intake (lesser half of cohort)*  *High intake*  Recurrence: MNVA |
| Mitra et al. - 2013^[59]^ | Prospective cohort | 212 patients with NMIBC/MIBC | ACM: ND | 1987-1996 | 58.9 (median) | 13.2 years (median) | **Smoking**  *Ref. = Never or ≤20 cigarettes/day for ≤30 years*  *Smoking for 31-40 years or >20 cigarettes/day for ≤30 years*  ACM: 2.59 (1.29-5.21)  *Smoking for >40 years*  ACM: 6.11 (3.02-  12.37) |
| Naito et al. - 2008^[60]^ | Randomized controlled trial | 202 patients with NMIBC | Recurrence: Positive findings on cystoscopy or consecutive positive findings on urine cytology.  Progression: MIBC or metastasis.  CSM/ACM: ND | 1999-2002 | Mean/median NR | 26.9-43.6 months (median) | ***Lactobacillus casei* probiotic**  *Ref. = No use*  Recurrence: 0.5654 (0.3450-0.9265)  Progression: NS on univariable analysis  CSM: NS on univariable analysis  ACM: NS on univariable analysis  **Smoking**  *Ref. = Non-smoker*  Recurrence: NS on univariable analysis |
| Nayan et al. - 2015^[61]^ | Retrospective cohort | 85 patients with NMIBC/MIBC | Recurrence: Imaging, cystoscopic, or physical examination evidence of disease recurrence.  CSM/ACM: Obtained through electronic medical record review and the Princess Margaret Hospital Cancer Registry. | 1997-2013 | 71 (mean) | 50 months (median) | **Metformin**  *Ref. = No use*  Recurrence: 0.38 (0.20-0.72)  CSM: 0.57 (0.35-0.91)  ACM: 1.05 (0.49-2.26)  **Other oral hypoglycemics**  *Ref. = No use*  Recurrence: 1.00 (0.57-1.76)  CSM: 0.65 (0.42-1.02)  ACM: 0.96 (0.46-1.98)  **Insulin**  *Ref. = No use*  Recurrence: 0.96 (0.22-4.10)  CSM: 0.56 (0.12-2.60)  ACM: 0.60 (0.21-1.72) |
| Nepple et al. - 2010^[62]^ | Randomized controlled trial | 670 patients with NMIBC | Recurrence: Confirmed by biopsy or cytology. | 1999-2003 | 68.4 (mean) | 24 months (median) | **Megadose multivitamins**  *Ref. = Recommended daily allowance*  Recurrence: 1.07 (0.83-1.39) |
| Nerli et al. - 2018^[63]^ | Retrospective cohort | 42 patients with NMIBC | Recurrence: Occurrence of a new tumor in the bladder. | 2007-2016 | 57.3 (mean) | 57.38 (median) | **Smoking*****  *Ref. = Tobacco non-users*  *Tobacco users*  Recurrence: Significantly higher. HR for multivariable analysis NR. |
| Newling et al. - 1995^[64]^ | Randomized controlled trial | 252 patients with NMIBC | Recurrence: Identification on cystoscopy.  Progression: ND  ACM: ND | 1979-1981 | 65 (median) | 3.4 years (mean) | **Pyridoxine**  *Ref. = Placebo*  Recurrence: NS on univariable analysis  Progression: NS on univariable analysis  ACM: NS on univariable analysis |
| Ogihara et al. - 2016^[65]^ | Retrospective cohort | 634 patients with NMIBC | Recurrence: ND | 1995-2012 | 68.5 (mean) | 68.1 months (median) | **Smoking**  *Ref. = Non-smoker*  Recurrence: 2.55 (1.70-3.83)  **Smoking – cumulative**  *Ref. = <1 pack/day*  ≥*1 pack/day*  Recurrence: NS on univariable analysis  **Smoking – cumulative**  *Ref. = <30 years*  ≥*30 years*  Recurrence: NS on univariable analysis  **Smoking – cumulative**  *Ref. = Quit* ≥*15 years prior to initial consultation*  *Quit <15 years prior to initial consutlation*  Recurrence: 2.20 (1.31-3.70) |
| P’ng et al. - 1993^[66]^ | Retrospective cohort | 45 patients with NMIBC | Therapeutic failure: persistent or recurrent tumor(s), or positive cytology at the first follow-up cystoscopy at 3 months. | 1985-1990 | 69 (mean), 70 (median) | 20.3 months (median) | **FCI (aspirin, NSAIDs, warfarin)**  *Ref. = No use*  Therapeutic failure (including recurrence): MVNA |
| Pastore et al. - 2015^[67]^ | Retrospective cohort | 574 patients with NMIBC | Recurrence: According to the American Cancer Society as the return of cancer after treatment and after a period of time during which the cancer could be detected, and at the site where it began (somewhere else in the bladder or at distant sites). | 2008-2013 | 62.24 (mean) | 45.06 months (mean) | **Aspirin**  *Ref. = No use*  Recurrence: 0.749 (0.452-1.239)  **Aspirin and statins**  *Ref. = No use*  Recurrence: 1.394 (0.852-2.279)  **Smoking**  *Ref. = Never*  *Former*  Recurrence: 2.191 (1.382-3.478)  *Current*  Recurrence: 3.202 (1.983-5.171)  **Statins**  *Ref. No use*  Recurrence: 1.886 (1.095-3.247) |
| Pedersen et al. - 1984^[68]^ | Randomized controlled trial | 73 patients with NMIBC | Recurrence: ND | NR | NR | 8 months (study period) | **Etretinate**  *Ref. = Placebo*  Recurrence: NS on univariable analysis |
| Psutka et al. - 2014^[69]^ | Retrospective cohort | 205 patients with NMIBC/MIBC | CSM/ACM: Cause of death is confirmed via death certificate. | 2000-2007 | 71 (median) | 6.7 years (median) | **BMI**  *Continuous*  CSM: 1.00 (0.97-1.05)  ACM: 1.00 (0.96-1.03) |
| Psutka et al. - 2015^[70]^ | Retrospective cohort | 262 patients with NMIBC/MIBC | ACM: Verified via death certificate. | 2000-2008 | 71 (median) | 6.3 years (median) | **BMI**  *Ref. = <30*  ≥*30*  ACM: 0.79 (0.50-1.26)  **Smoking**  *Ref. = Non-smoker*  ACM: 1.24 (0.81-1.92) |
| Raitanen et al. - 1995^[71]^ | ND | 169 patients NMIBC/MIBC | Recurrence: ND | 1978-1986 | 65-67 (mean) | 7.5 years (mean) | **Smoking**  *Ref. = Non-smoker*  Recurrence: NS on univariable analysis |
| Raitanen et al. - 1995^[72]^ | ND | 252 patients with NMIBC/MIBC | CSM: ND | 1978-1986 | 63-66 (mean) | 6.7 years (mean) | **Smoking**  *Ref. = Non-smoker*  *Smoker*  CSM: 1.4 (0.9-2.3) |
| Rausch et al. - 2014^[73]^ | Retrospective cohort | 192 patients with NMIBC | Recurrence: Tumor recurrence with or without pathological upstaging or upgrading.  Progression: Pathological progression by either upstaging or upgrading. Occurrence of a staging of greater or equal to pT2. | 1996-2006 | 68.31 (median) | 80 months (median) | **Smoking**  *Ref. = Non-smoker*  Recurrence: NS on univariable analysis  Progression: NS on univariable analysis  **DMII**  *Ref. = No DMII*  Recurrence: NS on univariable analysis  Progression: NS on univariable analysis |
| Richard et al. - 2017^[74]^ | Retrospective cohort | 13,811 patients with NMIBC | CSM/ACM: As indicated in databases. | 1992-2002 | 76 (median) | 7.1 years (median) | **Statins**  *Ref. = No use*  *Cumulative use before diagnosis*  CSM: 0.99 (0.97-1.01)  ACM: 1.01 (0.99-1.03)  *Cumulative use after diagnosis*  CSM: 1.04 (0.99-1.09)  ACM: 0.93 (0.91-0.96)  **DMII**  *Ref. = No DMII*  CSM: 0.99 (0.97-1.01)  ACM: 1.04 (1.03-1.05) |
| Richard et al. - 2018^[75]^ | Retrospective  cohort | 1,742 patients with NMIBC | CSM/ACM: As indicated in databases. | 1992-2012 | 78 (median) | 5.2 years (median) | **DMII**  *Cumulative time between DMII and NMIBC diagnosis*  CSM: 1.0 (0.96-1.1)  ACM: 1.01 (0.99-1.03)  **Glyburide**  *Ref. = No use*  *Cumulative use before diagnosis*  CSM: 0.97 (0.88-1.1)  ACM: 1.06 (1.02-1.1)  *Cumulative use after diagnosis*  CSM: 1.17 (1.02-1.3)  ACM: 1.01 (0.97-1.1)  **Insulin**  *Ref. = No use*  *Cumulative use before diagnosis*  CSM: 1.1 (0.65-1.9)  ACM: 1.3 (1.11-1.5)  *Cumulative use after diagnosis*  CSM: 1.17 (0.76-1.8)  ACM: 1.09 (0.87-1.2)  **Metformin**  *Ref. = No use*  *Cumulative use before diagnosis*  CSM: 1.0 (0.90-1.1)  ACM: 1.0 (0.97-1.1)  *Cumulative use after diagnosis*  CSM: 1.1 (0.92-1.2)  ACM: 0.96 (0.92-1.01)  **Other oral anti-diabetic agents**  *Ref. = No use*  *Cumulative use before diagnosis*  CSM: 0.99 (0.70-1.4)  ACM: 0.97 (0.86-1.1)  *Cumulative use after diagnosis*  CSM: 0.86 (0.53-1.4)  ACM: 1.06 (0.85-1.2)  **Thiazolidinedione**  *Ref. = No use*  *Cumulative use before diagnosis*  CSM: 0.85 (0.36-2.0)  ACM: 0.1 (0.83-1.1)  *Cumulative use after diagnosis*  CSM: 0.85 (0.30-1.3)  ACM: 0.91 (0.77-1.1) |
| Rieken et al. - 2013^[76]^ | Retrospective cohort | 1,117 patients with NIMBC | Recurrence: First tumor relapse in the bladder or prostatic urethra, regardless of tumor stage.  Progression: Tumor relapse at tumor stage T2 or higher in the bladder or prostatic urethra.  CSM/ACM: The cause of death was determined by the treating physicians, by chart review corroborated by death certificates, or by death certificates alone. | 1996-2007 | 65 (mean), 67 (median) | 64 months (median) | **Smoking**  *Ref. = Never*  *Former*  Recurrence: NS on univariable analysis  Progression: NS on univariable analysis  CSM: NS on univariable analysis  ACM: NS on univariable analysis  *Current*  Recurrence: NS on univariable analysis  Progression: MVNA  CSM: NS on univariable analysis  ACM: NS on univariable analysis  **DMII**  *Ref. = No DMII*  *DMII, no metformin*  Recurrence: 1.39 (1.04-1.86)  Progression: 2.21 (1.29-3.77)  CSM: NS on univariable analysis  ACM: 1.19 (0.81-1.73)  *DMII, metformin*  Recurrence: 0.48 (0.26-0.89)  Progression: 0.34 (0.05-2.42)  CSM: NS on univariable analysis  ACM: 1.49 (0.88-2.53) |
| Rieken et al. - 2014^[77]^ | Retrospective cohort | 1,502 patients with NMIBC/MIBC | Recurrence: Tumor relapse in the operative field, regional lymph nodes, and distant metastases.  CSM/ACM: Cause of death was determined by treating physicians by chart review corroborated by death certificates or by death certificates alone. | 1992-2008 | 65.5 (mean), 66 (median) | 34 months (median) | **BMI**  *Continuous*  Recurrence: 1.05 (1.03-1.07)  CSM: 1.05 (1.03-1.07)  ACM: 1.01 (0.99-1.03)  **DMII**  *Ref. = No DMII*  *DMII, no metformin*  Recurrence: 1.29 (0.97-1.73)  CSM: 1.53 (1.12-2.09)  ACM: 1.52 (1.16-2.00)  *DMII, metformin*  Recurrence: 0.96 (0.63-1.46)  CSM: 1.01 (0.62-1.63)  ACM: 0.99 (0.65-1.50)  **Smoking**  *Ref. = Non-smoker*  Recurrence: 1.19 (1.05-1.35)  CSM: 1.17 (1.02-1.35)  ACM: 1.10 (0.98-1.24) |
| Rink et al. - 2012^[78]^ | Retrospective cohort | 390 patients with NMIBC | Recurrence: First tumor relapse in the bladder regardless of stage.  Progression: Tumor relapse with an increase to disease stage T2 or higher in the bladder.  ACM: ND | 1987-2007 | 67 (median) | 66 months (median) | **Smoking (categorical)**  *Ref. = Never*  *Former*  Recurrence: NS on univariable analysis Progression: NS on univariable analysis  ACM: NS on univariable analysis  *Current*  Recurrence: NS on univariable analysis Progression: NS on univariable analysis  ACM: NS on univariable analysis  **Smoking (cessation)**  *Ref. = Current*  *Cessation* ≥*10 years*  Recurrence: 0.403 (0.241-0.671)  Progression: 0.509 (0.223-1.161)  *Cessation <10 years*  Recurrence: 1.438 (0.995-2.079)  Progression: 1.263 (0.666-2.393)  **Smoking (cumulative)**  *Ref. = Light short-term*  *Moderate*  Recurrence: 2.075 (1.231-3.496)  *Heavy long-term*  Recurrence: 4.307 (2.434-7.622) |
| Rink et al. - 2012^[79]^ | Retrospective cohort | 1,987 patients with NMIBC | Recurrence: First tumor relapse in the bladder regardless of tumor stage. | 1987-2007 | 68 (median) | NR | **Smoking**  *Risk of recurrence greater among BCG treated smokers (HR 1.44, 95% CI 1.01-2.04). NS risk of recurrence between those who did versus did not receive BCG among former and never smokers.* |
| Rink et al. - 2013^[80]^ | Retrospective cohort | 2,043 patients with NMIBC | Recurrence: First tumor relapse in the bladder regardless of tumor stage.  Progression: Tumor relapse at tumor stage T2 or higher in the bladder.  ACM: ND | 1987-2007 | 67 (median) | 49 months (median) | **Smoking (categorical)**  *Ref. = Never*  *Former*  Recurrence: 1.12 (0.94-1.34)  Progression: 1.29 (0.79-2.09)  ACM: 1.10 (0.86-1.41)  *Current*  Recurrence: 1.22 (1.01-1.48)  Progression: 2.09 (1.29-3.39)  ACM: 1.12 (0.85-1.47)  **Smoking (cessation)**  *Ref. = Current*  *Former <10 years*  Recurrence: 1.30 (1.09-1.53)  Progression: 0.99 (0.65-1.50)  ACM: 1.02 (0.79-1.30)  *Former* ≥*10 years*  Recurrence: 0.66 (0.52-0.84)  Progression: 0.42 (0.22-0.83)  ACM: 0.98 (0.72-1.34)  **Smoking (cumulative)**  *Ref. = Heavy long*  *Light long*  Recurrence: 0.91 (0.77-1.07)  Progression: 0.43 (0.29-0.63)  ACM: 0.67 (0.52-0.85)  *Heavy short*  Recurrence: 0.43 (0.30-0.60)  Progression: 0.12 (0.03-0.44)  ACM: 0.81 (0.51-1.27)  *Light short*  Recurrence: 0.35 (0.26-0.47)  Progression: 0.05 (0.01-0.19)  ACM: 0.54 (0.37-0.80) |
| Sabichi et al. - 2008^[81]^ | Randomized controlled trial | 137 patients with NMIBC | Recurrence: Cystoscopy, biopsy confirmation. | 1998-2003 | 64.5-69.2 (mean), 64.0-70.9 (median) | NR | **Fenretinide**  *Ref. = Placebo*  Recurrence: NS on univariable analysis |
| Sabichi et al. - 2011^[82]^ | Randomized controlled trial | 146 patients with NMIBC | Recurrence: A biopsy that yielded a histologically confirmed recurrence. | 2000-2005 | NR | 2.49 years (median) | **Celecoxib**  *Ref. = Placebo*  Recurrence: 0.69 (0.37-1.29) |
| Segal et al. - 2014^[83]^ | Retrospective cohort | 278 patients with NMIBC | Recurrence: ND  Worsening: Includes evidence of disease stage progression, need for radical cystectomy or disease-specific mortality. | 1995-2005 | 72.8 (median) | 3 years (median) | **Statins**  *Ref. = No use*  Recurrence: NS on univariable analysis  Worsening (includes progression and CSM): 0.784 (0.453-1.341)  **Smoking**  *Ref. = Non-smoker*  Recurrence: NS on univariable analysis Worsening (includes progression and CSM): NS on univariable analysis |
| Selinksi et al. - 2016^[84]^**** | Case-control | 795 patients with NMIBC | Recurrence: ND | 2008-2014 | 70.27 (median) | NR | **Aromatic amines**  *Ref. = No exposure*  *Exposure*  Recurrence: 1.13 (0.77-1.68)  **Chemical industry**  *Ref. = No exposure*  *Exposure*  Recurrence: 1.29 (0.86-1.94)  **Painter/varnisher**  *Ref. = No exposure*  *Exposure*  Recurrence: 0.70 (0.36-1.37) |
| Serretta et al. - 2013^[85]^ | Retrospective cohort | 395 patients with NMIBC | Recurrence: Pathologically confirmed | 2002-2003 | 68 (median) | 48 months (median) | **Smoking (binary)**  *Ref. = Non-smoker*  Recurrence: 1.60 (1.02-2.50)  **Smoking (categorical)**  *Ref. = Never*  *Former*  Recurrence: 1.94 (1.18-3.18)  *Current*  Recurrence: 1.39 (0.40-2.24)  ***Smoking (categorical)***  *Ref. = Former*  *Current*  Recurrence: NS on univariable analysis |
| Sfakianos et al. - 2011^[86]^ | Retrospective cohort | 623 patients with NMIBC | Recurrence: Visual and/or biopsy-confirmed evidence of tumor at cystoscopy or positive urine cytology.  Progression: The development of an invasive tumor of higher stage or by the presence of metastatic disease.  CSM/ACM: The cause of death was determined by  the treating physicians or by chart review corroborated by death certificates. | 1994-2008 | 75 (mean), 76 (median) | 80.9 months (median) | **Smoking (binary)**  *Ref. = Never*  *Smoker*  Recurrence: 1.05 (0.84-1.31)  Progression: 1.02 (0.66-1.59) CSM: 1.15 (0.68-1.96)  ACM: 1.14 (0.79-1.64)  **Smoking (categorical)**  *Ref. = Never*  *Former*  Recurrence: 1.05 (0.84-1.32)  Progression: 1.00 (0.64-1.58)  CSM: 1.14 (0.66-1.97)  ACM: 1.20 (0.82-1.74)  *Current*  Recurrence: 1.04 (0.77-1.40)  Progression: 1.16 (0.65-2.10)  CSM: 1.27 (0.64-2.53)  ACM: 1.03 (0.63-1.68)  **Smoking (categorical)**  *Ref. = Never*  *Stopped >10 years*  Recurrence: 1.06 (0.83-1.35)  Progression: 1.06 (0.65-1.72)  CSM: 1.29 (0.72-2.29)  ACM: 1.34 (0.90-1.98)  *Stopped 0.1-10 years*  Recurrence: 1.22 (0.90-1.66)  Progression: 0.95 (0.51-1.77)  CSM: 0.96 (0.45-2.06)  ACM: 1.16 (0.71-1.90)  *Stopped at diagnosis*  Recurrence: 0.75 (0.49-1.16)  Progression: 0.81 (0.35-1.88)  CSM: 0.80 (0.30-2.18)  ACM: 0.64 (0.31-1.34)  *Current*  Recurrence: 1.04 (0.77-1.40)  Progression: 1.16 (0.65-2.08)  CSM: 1.27 (0.64-2.52)  ACM: 1.03 (0.63-1.68) |
| Singla et al. – 2017^[87]^ | Prospective cohort | 99 patients with NMIBC | Recurrence: ND  Progression: Stage, including development of muscle-invasive (≥pT2) or metastatic disease. Progression to radical cystectomy was also evaluated.  CSM/ACM: ND | 2006-2012 | 73 (median) | 31.4 months (median) | **BMI**  *Continuous*  Recurrence: NS on univariable analysis  Stage progression: NS on univariable analysis  Progression to cystectomy: NS on univariable analysis  CSM: NS on univariable analysis  ACM: 0.86 (0.76–0.96)  **Smoking*****  *Ref. = Non-smoker*  Recurrence: NS on univariable analysis  Stage progression: NS on univariable analysis  Progression to cystectomy: NS on univariable analysis  CSM: NS on univariable analysis  ACM: NS on univariable analysis  **Aspirin use**  *Ref. = No use*  Recurrence: NS on univariable analysis  Stage progression: NS on univariable analysis  Progression to cystectomy: NS on univariable analysis  CSM: NS on univariable analysis  ACM: NS on univariable analysis  **NSAID or COX inhibitor use**  *Ref. = No use*  Recurrence: NS on univariable analysis  Stage progression: NS on univariable analysis  Progression to cystectomy: NS on univariable analysis  CSM: NS on univariable analysis  ACM: NS on univariable analysis  **Statin**  *Ref. = No use*  Recurrence: NS on univariable analysis  Stage progression: NS on univariable analysis  Progression to cystectomy: NS on univariable analysis  CSM: NS on univariable analysis  ACM: NS on univariable analysis |
| Skolarus et al. - 2009^[88]^ | Retrospective cohort | 90 patients with NMIBC | Recurrence: Total recurrences.  Progression: Local tumor progression; undergoing cystectomy, chemotherapy or radiation therapy; or development of metastatic disease.  CSM/ACM: ND | 1997-2007 | 68.1-68.9 (mean) | 5.1 years (median) | **Statins**  *Ref. = No use*  Recurrence: NS on univariable analysis  Progression: NS on univariable analysis  CSM: NS on univariable analysis  ACM: NS on univariable analysis |
| Studer et al. - 1995^[89]^ | Randomized controlled trial | 79 patients with NMIBC | Recurrence: First recurrence on cystoscopy. | NR | 59.5 (mean) | 30-33 months (median) | **Etretinate:**  *Ref. = Placebo*  Recurrence: NS on univariable analysis |
| Takashi et al. - 1987^[90]^ | ND | 264 patients with NMIBC/MIBC | ACM: ND | 1973-1984 | Mean/median NR | NR | **Smoking**  *Ref. = Non-smoker*  ACM: NS on univariable analysis |
| Thompson et al. - 1987^[91]^ | Retrospective cohort | 368 patients with NMIBC/MIBC | Recurrence: ND | 1980-1985 | 57.3-60.7 (mean) | NR | **Smoking**  *Ref. = Non-smoker*  Recurrence: MVNA |
| Tang et al. - 2010^[92]^**** | Retrospective cohort | 239 patients with NMIBC/MIBC | CSM: Death attributable to bladder cancer as a primary or underlying cause on the death certificate.  ACM: Death from any cause including bladder cancer. | 1980-1998 | Mean/median NR | 96 months (mean) | **Broccoli, cooked**  *Ref. = <1 serving/month*  ≥*1 serving/month*  CSM: 0.68 (0.45-1.01)  ACM: 0.67 (0.49-0.91)  **Broccoli, raw**  *Ref. = <1 serving/month*  *1 serving/month*  CSM: 0.43 (0.25-0.74)  ACM: 0.57 (0.39-0.83)  **Fruit**  *Ref. = <27.5 servings/month*  *27.5-51 servings/month*  CSM: 0.94 (0.57-1.55)  ACM: 0.86 (0.59-1.24)  *>51 servings/month*  CSM: 1.09 (0.66-1.81)  ACM: 0.91 (0.62-1.33)  **Raw Cruciferous**  *Ref. = <1 servings/month*  *1-3 servings/month*  CSM: 0.67 (0.41-1.10)  ACM: 0.67 (0.46-0.97)  *>3 servings/month*  CSM: 0.73 (0.44-1.21)  ACM: 0.73 (0.50-1.06)  **Vegetables**  *Ref. = <52 servings/month*  *52-85.5 servings/month*  CSM: 0.95 (0.59-1.55)  ACM: 0.90 (0.62-1.30)  *>85.5 servings/month*  CSM: 1.06 (0.63-1.78)  ACM: 0.91 (0.62-1.36) |
| Tu et al. - 2018^[93]^ | Prospective cohort | 619 patients with NMIBC | Recurrence: Newly found bladder tumor.  Progression: The transition from NMIBC to MIBC or metastatic disease. | 1995-2003 | 61.7-64.8 (mean) | 62 months (median) | **Folate (natural)**  *Ref. = Low (tertile 1)*  *Medium (tertile 2)*  Recurrence: 0.96 (0.71-1.30)  Progression: 0.67 (0.40-1.11)  *High (tertile 3)*  Recurrence: 0.82 (0.60-1.13)  Progression: 0.69 (0.41-1.15)  **Folate (synthetic)**  *Ref. = Low (tertile 1)*  *Medium (tertile 2)*  Recurrence: 1.72 (1.20-2.48)  Progression: 1.17 (0.65-2.12)  *High (tertile 3)*  Recurrence: 1.80 (1.14-2.84)  Progression: 1.33 (0.63-2.81)  **Folate (total)**  *Ref. = Low (tertile 1)*  *Medium (tertile 2)*  Recurrence: 1.67 (1.16-2.38)  Progression: 1.83 (1.02-3.26)  *High (tertile 3)*  Recurrence: 1.23 (0.78-1.95)  Progression: 1.20 (0.57-2.53) |
| Van Osch et al. - 2016^[94]^ | Meta-analysis | 6908 patients with NMIBC from 11 studies | Recurrence: Local recurrence.  Progression: ND  CSM: ND | 1995-2014 | Mean/median NR | 14-81 months (median) | **Smoking**  *Ref. = Never*  *Former (5382 patients)*  Recurrence: 1.13 (1.00-1.25)  Progression: 1.13 (0.81-1.45)  *Current*  Recurrence: 1.27 (1.09-1.46)  Progression: 1.21 (0.81-1.61)  CSM (925 patients): 1.01 (0.93-1.10) |
| Van Osch et al. - 2018^[95]^**** | Prospective cohort | 722 patients with NMIBC | Recurrence: A new tumor that was at the same stage as the primary tumor (Ta or T1) but also when a primary Ta patient had a T1 recurrence. | 2005-2011 | 71 (median) | 4.21 years (median) | **Smoking (categorical)**  *Ref. = Never*  *Former*  Recurrence: 0.78 (0.48-1.24)  *Current*  Recurrence: 1.04 (0.65-1.66)  *Former who started again*  Recurrence: 0.87 (0.53-1.41)  *Current who quit*  Recurrence: 1.47 (0.63-3.41)  **Smoking (cessation)**  *Ref. = Current*  *<20 years*  Recurrence: 0.82 (0.46-1.46)  *21-40 years*  Recurrence: 0.74 (0.51-1.08)  *>40 years*  Recurrence: 0.71 (0.46-1.09) |
| Wakai et al. - 1993^[96]^**** | Prospective cohort | 258 patients with NMIBC/MIBC | ACM: ND | 1976-1978 | Mean/median NR | 29.8 months (median) | **Alcohol (Males only)**  *Ref. = Never*  *Ex-drinker*  ACM: 0.60 (0.23-1.59)  *Current (<2 gou/day)*  ACM: 0.41 (0.22-0.77)  *Current (2-4 gou/day)*  ACM: 0.43 (0.16-1.14)  *Current (*≥*4 gou/day)*  ACM: 0.82 (0.22-3.13)  **Hair dye**  *Ref. = No exposure*  *Exposure*  ACM: 0.65 (0.28-1.79)  **Smoking**  *Ref. = Non-smoker*  ACM: 0.88 (0.45-1.72) |
| Westhoff et al. - 2018^[97]^**** | Prospective cohort | 595 patients with NMIBC | Recurrence: New bladder tumor following a previous negative follow-up cystoscopy.  Progression: The transition from NMIBC to muscle-invasive or metastatic tumors. | 1995 “onward” | 62.5-66.0 (mean) | 65.7 months (median) | **Fruits and vegetables**  *Ref. = Tertile 1*  *Tertile 2*  Recurrence: 1.08 (0.79-1.47)  Progression: 1.16 (0.70-1.92)  *Tertile 3*  Recurrence: 0.90 (0.64-1.26)  Progression: 1.05 (0.61-1.82)  **Low-fat pattern**  *Ref. = Tertile 1*  *Tertile 2*  Recurrence: 0.96 (0.71-1.31)  Progression: 0.78 (0.47-1.28)  *Tertile 3*  Recurrence: 0.86 (0.63-1.18)  Progression: 0.74 (0.44-1.23)  **Tex-Mex pattern**  *Ref. = Tertile 1*  *Tertile 2*  Recurrence: 1.08 (0.79-1.48)  Progression: 1.27 (0.79-2.06)  *Tertile 3*  Recurrence: 0.92 (0.66-1.27)  Progression: 0.70 (0.40-1.24)  **Western diet pattern**  *Ref. = Tertile 1*  *Tertile 2*  Recurrence: 1.03 (0.75-1.42)  Progression: 1.23 (0.73-2.06)  *Tertile 3*  Recurrence: 1.48 (1.06-2.06)  Progression: 1.56 (0.91-2.65) |
| Westhoff et al. - 2018^[98]^ | Meta-analysis | 1,633 patients with NMIBC from three studies and 5,533 patients with NMIBC/MIBC from three studies | Recurrence: ND  Progression: ND  CSM/ACM: ND | Up to 2017 | Mean/median NR | NR | **BMI – NMIBC**  *Ref. = Normal weight*  *Overweight*  Recurrence: 1.29 (1.05-1.58)  Progression (2 studies, 1,294 patients): 1.03 (0.63-1.70)  *Obese*  Recurrence: 1.82 (1.12-2.95)  Progression (2 studies, 1,294 patients): 1.90 (0.93-3.88)  **BMI – NMIBC**  *Ref. = Normal weight*  *Overweight*  Recurrence: 0.87 (0.67-1.14)  CSM: 0.82 (0.65-1.02)  *Obese*  Recurrence: 1.12 (0.54-2.31)  CSM: 0.98 (0.46-2.10) |
| Witjes et al. - 1993^[99]^ | Prospective cohort | 183 patients with NMIBC | Recurrence: Positive cystoscopy, cytology, or biopsy. | 1987-1991 | Mean/median NR | 20.8-22.5 months (median) | **FCIs (aspirin, dipyramidole, ibuprofen, indomethacin, naproxen, warfarin)**  *Ref. = No use*  Recurrence: NS on univariable analysis. |
| Wyszynski et al. - 2014^[100]^ | ND | 726 patients with NMIBC | Recurrence: Any tumor identified after a disease-free remission period, more than 90 days after the date of initial primary bladder tumor diagnosis.  Progression: The diagnosis of a tumor with a greater stage or grade than the initial primary bladder tumor.  ACM: Life status (alive or deceased) was determined as of January 2011 using the Social Security and the National Death Indices (NDI). | 1994-2001 | Mean/median NR | 6 years (median) | **BMI**  *Ref. = ≤24.9*  *24.9-29.9*  Recurrence: 1.39 (0.96-2.01)  ≥*30*  Recurrence: 1.22 (0.80-1.87)  ≥*24.9*  Recurrence: 1.33 (0.94-1.89)  **Smoking**  *Ref. = Never*  *Former*  Recurrence: 1.61 (1.17-2.20)  Progression: NS  ACM: 1.69 (0.70-4.10)  *Current*  Recurrence: 1.51 (1.08–2.13)  Progression: NS  ACM: 3.42 (1.29-9.07)  *Quit* ≥*29 years prior*  Recurrence: 1.37 (0.89-2.10)  *Quit 19-28 years prior*  Recurrence: 1.44 (0.94–2.21)  *Quit 1-8 years prior*  Recurrence: 1.83 (1.30-2.59) |
| Xu et al. - 2015^[101]^ | Retrospective cohort | 403 patients with NMIBC | Recurrence: The first tumor relapse in the bladder.  Progression: An increase to pathologic T2 stage or higher in the bladder. | 2006-2014 | 67.1 (mean) | 53 months (median) | **BMI**  *Ref. = <24*  *24-<28*  Recurrence: 1.435 (1.029-2.002)  Progression: 1.362 (0.571-3.249)  *>28*  Recurrence: 1.707 (1.120-2.602)  Progression: 3.037 (1.243-7.420)  **DMII**  *Ref. = No DMII*  Recurrence: 1.803 (1.141-2.850)  Progression: 3.111 (1.113-8.696) |
| Yafi et al. - 2011^[102]^ | Retrospective cohort | 2287 patients with NMIBC/MIBC | Recurrence/progression: Local (pelvic) and/or distant (metastasis).  CSM/ACM: Cause of death was determined by the treating physician, based on chart review and/or the death certificate. | 1998-2008 | 68 (median) | 35 months (mean), 29 months (median) | **Smoking**  Recurrence/progression: MVNA  CSM: 1.304 (1.005-1.691)  ACM: 1.307 (1.049-1.628) |
| Yonekura et al. - 2018^[103]^ | Retrospective cohort | 50 patients with NMIBC | Recurrence: Histologically verified urothelial carcinoma involving any site within the bladder. | 2011-2016 | 73 years (median) | 38.55 months (median) | **BMI**  *Continuous*  Recurrence: 1.138 (1.021-1.268)  **Smoking**  *Ref. = Never*  *Current/former*  Recurrence: NS on univariable analysis |
| Yu et al. - 1997^[104]^ | Retrospective cohort | 870 patients with NMIBC/MIBC | ACM: ND | 1990-1995 | Bladder cancer-specific NR | Bladder cancer-specific NR | **Smoking**  ACM: RR 1.18 (Statistically insignificant, no p-value or confidence interval available) |
| Yuruk et al. - 2017^[105]^ | Retrospective cohort | 187 patients with NMIBC | Recurrence: ND | 2013-2014 | 64.68 (mean) | 32.28 months (mean) | **Smoking**  *Ref. = Never*  *Former*  Recurrence: NS on univariable analysis  *Current*  Recurrence: NS on univariable analysis |

ACM = All-cause mortality, BMI = Body mass index, CIS = Carcinoma in-situ, CSM = Cancer-specific mortality, CSS = Cancer-specific survival, DMII = Diabetes Mellitus Type 2, FCI = Fibrin Clot Inhibitor, MVNA = Multivariable analysis not available, MIBC = Muscle invasive bladder cancer, ND = Not defined, NMIBC = Non-muscle invasive bladder cancer, NR = Not reported, NS = No significance, OS = Overall survival, RFS = Recurrence-free survival, RR = Relative risk, SE = Standard error, SSRE = Summary relative risk estimate, UK = United Kingdom, US = United States

*Directly from Methods section of text or assessed to best ability. ND indicates insufficient information in text for certainty of designation/definition.

**Presented as Hazard Ratio (95% confidence interval) unless otherwise specified. For cohort studies, only results from multivariable analyses are shown.

***Smoking included in general variable of “tobacco use.”

****Only select items of interest are shown. See study for full list of evaluated factors.

**References**

1. Ahirwar, D., P. Kesarwani, P.K. Manchanda, et al., *Anti- and proinflammatory cytokine gene polymorphism and genetic predisposition: association with smoking, tumor stage and grade, and bacillus Calmette-Guerin immunotherapy in bladder cancer.* Cancer Genet Cytogenet, 2008. **184**(1): p. 1-8. DOI: 10.1016/j.cancergencyto.2008.02.015.

2. Ahn, J.H., S.I. Jung, S.U. Yim, et al., *Impact of Glycemic Control and Metformin Use on the Recurrence and Progression of Non-Muscle Invasive Bladder Cancer in Patients with Diabetes Mellitus.* Journal of Korean medical science, 2016. **31**(9): p. 1464-1471. DOI: 10.3346/jkms.2016.31.9.1464.

3. Ajili, F., N. Kourda, S. Karay, et al., *Impact of smoking intensity on outcomes of patients with non muscle invasive bladder cancer treated by BCG immunotherapy.* Ultrastruct Pathol, 2013. **37**(4): p. 273-7. DOI: 10.3109/01913123.2013.770111.

4. Alfthan, O., J. Tarkkanen, P. Grohn, et al., *Tigason (etretinate) in prevention of recurrence of superficial bladder tumors. A double-blind clinical trial.* Eur Urol, 1983. **9**(1): p. 6-9.

5. Allard, P., Y. Fradet, B. Tetu, et al., *Tumor-associated antigens as prognostic factors for recurrence in 382 patients with primary transitional cell carcinoma of the bladder.* Clin Cancer Res, 1995. **1**(10): p. 1195-202.

6. Aso, Y. and H. Akazan, *Prophylactic effect of a Lactobacillus casei preparation on the recurrence of superficial bladder cancer. BLP Study Group.* Urol Int, 1992. **49**(3): p. 125-9. DOI: 10.1159/000282409.

7. Aso, Y., H. Akaza, T. Kotake, et al., *Preventive effect of a Lactobacillus casei preparation on the recurrence of superficial bladder cancer in a double-blind trial. The BLP Study Group.* Eur Urol, 1995. **27**(2): p. 104-9.

8. Bachir, B.G., A.G. Aprikian, J.I. Izawa, et al., *Effect of body mass index on the outcomes of patients with upper and lower urinary tract cancers treated by radical surgery: results from a Canadian multicenter collaboration.* Urol Oncol, 2014. **32**(4): p. 441-8. DOI: 10.1016/j.urolonc.2013.10.016.

9. Berglund, R.K., C.J. Savage, K.C. Vora, et al., *An analysis of the effect of statin use on the efficacy of bacillus calmette-guerin treatment for transitional cell carcinoma of the bladder.* J Urol, 2008. **180**(4): p. 1297-300; discussion 1300. DOI: 10.1016/j.juro.2008.06.034.

10. Boorjian, S.A., R.K. Berglund, A.C. Maschino, et al., *Fibrin clot inhibitor medication and efficacy of bacillus Calmette-Guerin for bladder urothelial cancer.* J Urol, 2009. **182**(4): p. 1306-12. DOI: 10.1016/j.juro.2009.06.026.

11. Bostrom, P.J., T. Mirtti, J. Kossi, et al., *Twenty-year experience of radical cystectomy for bladder cancer in a medium-volume centre.* Scand J Urol Nephrol, 2009. **43**(5): p. 357-64. DOI: 10.3109/00365590902939387.

12. Byar, D. and C. Blackard, *Comparisons of placebo, pyridoxine, and topical thiotepa in preventing recurrence of stage I bladder cancer.* Urology, 1977. **10**(6): p. 556-61.

13. Cao, J., R. Xu, X. Zhao, et al., *Areca Nut Chewing and an Impaired Estimated Glomerular Filtration Rate as Significant Risk Factors for Non-Muscle-Invasive Bladder Cancer Recurrence.* Sci Rep, 2016. **6**: p. 29466. DOI: 10.1038/srep29466.

14. Carpenter, A.A., *Clinical Experience with Transitional Cell Carcinoma of the Bladder with Special Reference to Smoking.* The Journal of Urology, 1989. **141**(3, Part 1): p. 527-528. DOI: <https://doi.org/10.1016/S0022-5347(17)40880-9>.

15. Carta, A., S. Pavanello, G. Mastrangelo, et al., *Impact of Occupational Exposures and Genetic Polymorphisms on Recurrence and Progression of Non-Muscle-Invasive Bladder Cancer.* Int J Environ Res Public Health, 2018. **15**(8). DOI: 10.3390/ijerph15081563.

16. Chade, D.C., S.F. Shariat, G. Godoy, et al., *Clinical outcomes of primary bladder carcinoma in situ in a contemporary series.* J Urol, 2010. **184**(1): p. 74-80. DOI: 10.1016/j.juro.2010.03.032.

17. Chen, C.H., C.T. Shun, K.H. Huang, et al., *Stopping smoking might reduce tumour recurrence in nonmuscle-invasive bladder cancer.* BJU Int, 2007. **100**(2): p. 281-6; discussion 286. DOI: 10.1111/j.1464-410X.2007.06873.x.

18. Cheng, L., R.M. Neumann, A.L. Weaver, et al., *Predicting cancer progression in patients with stage T1 bladder carcinoma.* J Clin Oncol, 1999. **17**(10): p. 3182-7. DOI: 10.1200/jco.1999.17.10.3182.

19. Chromecki, T.F., E.K. Cha, H. Fajkovic, et al., *Obesity is associated with worse oncological outcomes in patients treated with radical cystectomy.* BJU Int, 2013. **111**(2): p. 249-55. DOI: 10.1111/j.1464-410X.2012.11322.x.

20. Crivelli, J.J., E. Xylinas, L.A. Kluth, et al., *Effect of statin use on outcomes of non-muscle-invasive bladder cancer.* BJU Int, 2013. **112**(2): p. E4-12. DOI: 10.1111/bju.12150.

21. da Silva, R.D., E. Xylinas, L. Kluth, et al., *Impact of statin use on oncologic outcomes in patients with urothelial carcinoma of the bladder treated with radical cystectomy.* J Urol, 2013. **190**(2): p. 487-92. DOI: 10.1016/j.juro.2013.02.003.

22. Dabi, Y., Y. Rouscoff, J. Anract, et al., *Impact of body mass index on the oncological outcomes of patients treated with radical cystectomy for muscle-invasive bladder cancer.* World J Urol, 2017. **35**(2): p. 229-235. DOI: 10.1007/s00345-016-1852-0.

23. Decensi, A., R. Torrisi, S. Bruno, et al., *Randomized trial of fenretinide in superficial bladder cancer using DNA flow cytometry as an intermediate end point.* Cancer Epidemiol Biomarkers Prev, 2000. **9**(10): p. 1071-8.

24. Donat, S.M., S. Bayuga, H.W. Herr, et al., *Fluid intake and the risk of tumor recurrence in patients with superficial bladder cancer.* J Urol, 2003. **170**(5): p. 1777-80. DOI: 10.1097/01.ju.0000091803.35049.da.

25. Ferro, M., M.D. Vartolomei, G.I. Russo, et al., *An increased body mass index is associated with a worse prognosis in patients administered BCG immunotherapy for T1 bladder cancer.* World J Urol, 2019. **37**(3): p. 507-514. DOI: 10.1007/s00345-018-2397-1.

26. Fleshner, N., J. Garland, A. Moadel, et al., *Influence of smoking status on the disease-related outcomes of patients with tobacco-associated superficial transitional cell carcinoma of the bladder.* Cancer, 1999. **86**(11): p. 2337-45.

27. Gee, J.R., D.F. Jarrard, R.C. Bruskewitz, et al., *Reduced bladder cancer recurrence rate with cardioprotective aspirin after intravesical bacille Calmette-Guerin.* BJU Int, 2009. **103**(6): p. 736-9. DOI: 10.1111/j.1464-410X.2008.08123.x.

28. Gierth, M., F. Zeman, S. Denzinger, et al., *Influence of Body Mass Index on Clinical Outcome Parameters, Complication Rate and Survival after Radical Cystectomy: Evidence from a Prospective European Multicentre Study.* Urol Int, 2018. **101**(1): p. 16-24. DOI: 10.1159/000488466.

29. Goossens, M.E., M.P. Zeegers, H. van Poppel, et al., *Phase III randomised chemoprevention study with selenium on the recurrence of non-invasive urothelial carcinoma. The SELEnium and BLAdder cancer Trial.* Eur J Cancer, 2016. **69**: p. 9-18. DOI: 10.1016/j.ejca.2016.09.021.

30. Grotenhuis, A.J., C.W. Ebben, K.K. Aben, et al., *The effect of smoking and timing of smoking cessation on clinical outcome in non-muscle-invasive bladder cancer.* Urol Oncol, 2015. **33**(2): p. 65.e9-17. DOI: 10.1016/j.urolonc.2014.06.002.

31. Hoffmann, P., T. Roumeguere, C. Schulman, et al., *Use of statins and outcome of BCG treatment for bladder cancer.* N Engl J Med, 2006. **355**(25): p. 2705-7. DOI: 10.1056/NEJMc062714.

32. Holz, S., S. Albisinni, J. Gilsoul, et al., *Risk factor assessment in high-risk, bacillus Calmette-Guerin-treated, non-muscle-invasive bladder cancer.* Res Rep Urol, 2017. **9**: p. 195-202. DOI: 10.2147/rru.S143865.

33. Hou, L., X. Hong, M. Dai, et al., *Association of smoking status with prognosis in bladder cancer: A meta-analysis.* Oncotarget, 2017. **8**(1): p. 1278-1289. DOI: 10.18632/oncotarget.13606.

34. Hudson, M.A., J.J. Yuan, W.J. Catalona, et al., *Adverse impact of fibrin clot inhibitors on intravesical bacillus Calmette-Guerin therapy for superficial bladder tumors.* J Urol, 1990. **144**(6): p. 1362-4.

35. Hwang, E.C., Y.J. Kim, I.S. Hwang, et al., *Impact of diabetes mellitus on recurrence and progression in patients with non-muscle invasive bladder carcinoma: a retrospective cohort study.* Int J Urol, 2011. **18**(11): p. 769-76. DOI: 10.1111/j.1442-2042.2011.02845.x.

36. Jochems, S.H.J., F.H.M. van Osch, R.C. Reulen, et al., *Total Fluid Intake and the Risk of Recurrence in Patients With Non-Muscle Invasive Bladder Cancer: A Prospective Cohort Study.* Bladder Cancer, 2018. **4**(3): p. 303-310. DOI: 10.3233/blc-180172.

37. Jochems, S.H.J., F.H.M. van Osch, R.C. Reulen, et al., *Fruit and vegetable intake and the risk of recurrence in patients with non-muscle invasive bladder cancer: a prospective cohort study.* Cancer Causes Control, 2018. **29**(6): p. 573-579. DOI: 10.1007/s10552-018-1029-9.

38. Kamat, A.M. and X. Wu, *Statins and the effect of BCG on bladder cancer.* N Engl J Med, 2007. **356**(12): p. 1276; author reply 1276-7. DOI: 10.1056/NEJMc070117.

39. Kashif Khan, M., I. Ahmed, and S.J. Raza, *Factors effecting recurrence and progression of high grade non invasive bladder cancer treated by intravesical BCG.* Pak J Med Sci, 2014. **30**(2): p. 326-30.

40. Kelly, J.D., W.S. Tan, N. Porta, et al., *BOXIT-A Randomised Phase III Placebo-controlled Trial Evaluating the Addition of Celecoxib to Standard Treatment of Transitional Cell Carcinoma of the Bladder (CRUK/07/004).* Eur Urol, 2019. **75**(4): p. 593-601. DOI: 10.1016/j.eururo.2018.09.020.

41. Kluth, L.A., E. Xylinas, J.J. Crivelli, et al., *Obesity is associated with worse outcomes in patients with T1 high grade urothelial carcinoma of the bladder.* J Urol, 2013. **190**(2): p. 480-6. DOI: 10.1016/j.juro.2013.01.089.

42. Koch, M., G.B. Hill, and M.S. McPhee, *Factors affecting recurrence rates in superficial bladder cancer.* J Natl Cancer Inst, 1986. **76**(6): p. 1025-9.

43. Korkes, F., C.A.B. Juliano, M.A.P. Bunduky, et al., *Amount of tobacco consumption is associated with superficial bladder cancer progression.* Einstein (São Paulo), 2010. **8**: p. 473-476.

44. Koshiaris, C., P. Aveyard, J. Oke, et al., *Smoking cessation and survival in lung, upper aero-digestive tract and bladder cancer: cohort study.* Br J Cancer, 2017. **117**(8): p. 1224-1232. DOI: 10.1038/bjc.2017.179.

45. Lacombe, L., V. Fradet, E. Levesque, et al., *Phase II Drug-Metabolizing Polymorphisms and Smoking Predict Recurrence of Non-Muscle-Invasive Bladder Cancer: A Gene-Smoking Interaction.* Cancer Prev Res (Phila), 2016. **9**(2): p. 189-95. DOI: 10.1158/1940-6207.Capr-15-0069.

46. Lamm, D.L., D.R. Riggs, J.S. Shriver, et al., *Megadose vitamins in bladder cancer: a double-blind clinical trial.* J Urol, 1994. **151**(1): p. 21-6.

47. Lammers, R.J., W.P. Witjes, K. Hendricksen, et al., *Smoking status is a risk factor for recurrence after transurethral resection of non-muscle-invasive bladder cancer.* Eur Urol, 2011. **60**(4): p. 713-20. DOI: 10.1016/j.eururo.2011.07.010.

48. Lee, C., K.H. Kim, D. You, et al., *Smoking and survival after radical cystectomy for bladder cancer.* Urology, 2012. **80**(6): p. 1307-12. DOI: 10.1016/j.urology.2012.08.026.

49. Leibovici, D., H.B. Grossman, C.P. Dinney, et al., *Polymorphisms in inflammation genes and bladder cancer: from initiation to recurrence, progression, and survival.* J Clin Oncol, 2005. **23**(24): p. 5746-56. DOI: 10.1200/jco.2005.01.598.

50. Lenis, A.T., K. Asanad, M. Blaibel, et al., *Association between Metabolic Syndrome and Recurrence of Nonmuscle Invasive Bladder Cancer following bacillus Calmette-Guérin Treatment.* Urology Practice, 2018. **5**(2): p. 132-138. DOI: <https://doi.org/10.1016/j.urpr.2017.02.012>.

51. Li, H.M., B. Azhati, M. Rexiati, et al., *Impact of smoking status and cumulative smoking exposure on tumor recurrence of non-muscle-invasive bladder cancer.* Int Urol Nephrol, 2017. **49**(1): p. 69-76. DOI: 10.1007/s11255-016-1441-6.

52. Lipsky, M.J., G.M. Badalato, P. Motamedinia, et al., *The effect of fibrin clot inhibitors on the immunomodulatory efficacy of Bacillus Calmette-Guerin therapy for non-muscle-invasive bladder cancer.* Urology, 2013. **81**(6): p. 1273-8. DOI: 10.1016/j.urology.2012.09.065.

53. Lopez-Beltran, A., G.A. Croghan, I. Croghan, et al., *Prognostic factors in survival of bladder cancer.* Cancer, 1992. **70**(4): p. 799-807.

54. Lukas, C., S. Selinski, H.M. Prager, et al., *Occupational bladder cancer: Polymorphisms of xenobiotic metabolizing enzymes, exposures, and prognosis.* J Toxicol Environ Health A, 2017. **80**(7-8): p. 439-452. DOI: 10.1080/10937404.2017.1304731.

55. Maurer, T., J. Maurer, M. Retz, et al., *Influence of body mass index on operability, morbidity and disease outcome following radical cystectomy.* Urol Int, 2009. **82**(4): p. 432-9. DOI: 10.1159/000218533.

56. Mazdak, H. and H. Zia, *Vitamin e reduces superficial bladder cancer recurrence: a randomized controlled trial.* International journal of preventive medicine, 2012. **3**(2): p. 110-115.

57. Michalek, A.M., K.M. Cummings, and J.E. Pontes, *Cigarette smoking, tumor recurrence, and survival from bladder cancer.* Prev Med, 1985. **14**(1): p. 92-8.

58. Michalek, A.M., K.M. Cummings, and J. Phelan, *Vitamin A and tumor recurrence in bladder cancer.* Nutr Cancer, 1987. **9**(2-3): p. 143-6. DOI: 10.1080/01635588709513921.

59. Mitra, A.P., J.E. Castelao, D. Hawes, et al., *Combination of molecular alterations and smoking intensity predicts bladder cancer outcome: a report from the Los Angeles Cancer Surveillance Program.* Cancer, 2013. **119**(4): p. 756-65. DOI: 10.1002/cncr.27763.

60. Naito, S., H. Koga, A. Yamaguchi, et al., *Prevention of recurrence with epirubicin and lactobacillus casei after transurethral resection of bladder cancer.* J Urol, 2008. **179**(2): p. 485-90. DOI: 10.1016/j.juro.2007.09.031.

61. Nayan, M., B. Bhindi, J.L. Yu, et al., *The effect of metformin on cancer-specific survival outcomes in diabetic patients undergoing radical cystectomy for urothelial carcinoma of the bladder.* Urol Oncol, 2015. **33**(9): p. 386.e7-13. DOI: 10.1016/j.urolonc.2015.05.024.

62. Nepple, K.G., A.J. Lightfoot, H.M. Rosevear, et al., *Bacillus Calmette-Guerin with or without interferon alpha-2b and megadose versus recommended daily allowance vitamins during induction and maintenance intravesical treatment of nonmuscle invasive bladder cancer.* J Urol, 2010. **184**(5): p. 1915-9. DOI: 10.1016/j.juro.2010.06.147.

63. Nerli, R.B., S.C. Ghagane, K. Shankar, et al., *Low-Grade, Multiple, Ta Non-muscle-Invasive Bladder Tumors: Tumor Recurrence and Worsening Progression.* Indian J Surg Oncol, 2018. **9**(2): p. 157-161. DOI: 10.1007/s13193-018-0728-8.

64. Newling, D.W., M.R. Robinson, P.H. Smith, et al., *Tryptophan metabolites, pyridoxine (vitamin B6) and their influence on the recurrence rate of superficial bladder cancer. Results of a prospective, randomised phase III study performed by the EORTC GU Group. EORTC Genito-Urinary Tract Cancer Cooperative Group.* Eur Urol, 1995. **27**(2): p. 110-6.

65. Ogihara, K., E. Kikuchi, K. Yuge, et al., *Refraining from Smoking for 15 Years or More Reduced the Risk of Tumor Recurrence in Non-muscle Invasive Bladder Cancer Patients.* Ann Surg Oncol, 2016. **23**(5): p. 1752-9. DOI: 10.1245/s10434-015-5016-z.

66. P'Ng K, B., M.D. Walsh, G.J. Seymour, et al., *The adverse effect of fibrin-clot inhibiting drugs on intravesical bacillus Calmette-Guerin efficacy for superficial bladder cancer.* Aust N Z J Surg, 1993. **63**(2): p. 127-30.

67. Pastore, A., G. Palleschi, A. Fuschi, et al., *Can daily intake of aspirin and/or statins influence the behavior of non-muscle invasive bladder cancer? A retrospective study on a cohort of patients undergoing transurethral bladder resection.* BMC Cancer, 2015. **15**: p. 120. DOI: 10.1186/s12885-015-1152-x.

68. Pedersen, H., H. Wolf, S.K. Jensen, et al., *Administration of a retinoid as prophylaxis of recurrent non-invasive bladder tumors.* Scand J Urol Nephrol, 1984. **18**(2): p. 121-3.

69. Psutka, S.P., A. Carrasco, G.D. Schmit, et al., *Sarcopenia in patients with bladder cancer undergoing radical cystectomy: impact on cancer-specific and all-cause mortality.* Cancer, 2014. **120**(18): p. 2910-8. DOI: 10.1002/cncr.28798.

70. Psutka, S.P., S.A. Boorjian, M.R. Moynagh, et al., *Mortality after radical cystectomy: impact of obesity versus adiposity after adjusting for skeletal muscle wasting.* J Urol, 2015. **193**(5): p. 1507-13. DOI: 10.1016/j.juro.2014.11.088.

71. Raitanen, M.P. and T.L. Tammela, *Impact of tumour grade, stage, number and size, smoking habits and sex on the recurrence rate and disease-free interval in patients with transitional cell carcinoma of the bladder.* Ann Chir Gynaecol, 1995. **84**(1): p. 37-41.

72. Raitanen, M.P., P. Nieminen, and T.L. Tammela, *Impact of tumour grade, stage, number and size, and smoking and sex, on survival in patients with transitional cell carcinoma of the bladder.* Br J Urol, 1995. **76**(4): p. 470-4.

73. Rausch, S., J. Hennenlotter, T. Todenhofer, et al., *Impaired estimated glomerular filtration rate is a significant predictor for non-muscle-invasive bladder cancer recurrence and progression--introducing a novel prognostic model for bladder cancer recurrence.* Urol Oncol, 2014. **32**(8): p. 1178-83. DOI: 10.1016/j.urolonc.2014.05.009.

74. Richard, P.O., A.E. Ahmad, S. Bashir, et al., *Effect of statins as a secondary chemopreventive agent among individuals with non-muscle-invasive bladder cancer: A population-based analysis.* Urol Oncol, 2017. **35**(6): p. 342-348. DOI: 10.1016/j.urolonc.2016.12.009.

75. Richard, P.O., A.E. Ahmad, S. Bashir, et al., *Impact of oral hypoglycemic agents on mortality among diabetic patients with non-muscle-invasive bladder cancer: A populationbased analysis.* Can Urol Assoc J, 2018. **12**(6): p. 203-210. DOI: 10.5489/cuaj.4870.

76. Rieken, M., E. Xylinas, L. Kluth, et al., *Association of diabetes mellitus and metformin use with oncological outcomes of patients with non-muscle-invasive bladder cancer.* BJU Int, 2013. **112**(8): p. 1105-12. DOI: 10.1111/bju.12448.

77. Rieken, M., E. Xylinas, L. Kluth, et al., *Effect of diabetes mellitus and metformin use on oncologic outcomes of patients treated with radical cystectomy for urothelial carcinoma.* Urol Oncol, 2014. **32**(1): p. 49.e7-14. DOI: 10.1016/j.urolonc.2013.07.006.

78. Rink, M., E. Xylinas, M. Babjuk, et al., *Impact of smoking on outcomes of patients with a history of recurrent nonmuscle invasive bladder cancer.* J Urol, 2012. **188**(6): p. 2120-7. DOI: 10.1016/j.juro.2012.08.029.

79. Rink, M., E. Xylinas, M. Babjuk, et al., *Smoking Reduces the Efficacy of Intravesical Bacillus Calmette-Guerin Immunotherapy in Non-muscle-invasive Bladder Cancer.* European Urology, 2012. **62**(6): p. 1204-1206. DOI: 10.1016/j.eururo.2012.08.057.

80. Rink, M., H. Furberg, E.C. Zabor, et al., *Impact of smoking and smoking cessation on oncologic outcomes in primary non-muscle-invasive bladder cancer.* Eur Urol, 2013. **63**(4): p. 724-32. DOI: 10.1016/j.eururo.2012.08.025.

81. Sabichi, A.L., S.P. Lerner, E.N. Atkinson, et al., *Phase III prevention trial of fenretinide in patients with resected non-muscle-invasive bladder cancer.* Clin Cancer Res, 2008. **14**(1): p. 224-9. DOI: 10.1158/1078-0432.Ccr-07-0733.

82. Sabichi, A.L., J.J. Lee, H.B. Grossman, et al., *A randomized controlled trial of celecoxib to prevent recurrence of nonmuscle-invasive bladder cancer.* Cancer Prev Res (Phila), 2011. **4**(10): p. 1580-9. DOI: 10.1158/1940-6207.Capr-11-0036.

83. Segal, R., F.A. Yafi, F. Brimo, et al., *Prognostic factors and outcome in patients with T1 high-grade bladder cancer: can we identify patients for early cystectomy?* BJU Int, 2012. **109**(7): p. 1026-30. DOI: 10.1111/j.1464-410X.2011.10462.x.

84. Selinski, S., H. Burger, M. Blaszkewicz, et al., *Occupational risk factors for relapse-free survival in bladder cancer patients.* J Toxicol Environ Health A, 2016. **79**(22-23): p. 1136-1143. DOI: 10.1080/15287394.2016.1219606.

85. Serretta, V., V. Altieri, G. Morgia, et al., *Cigarette smoking status at diagnosis and recurrence in intermediate-risk non-muscle-invasive bladder carcinoma.* Urology, 2013. **81**(2): p. 277-81. DOI: 10.1016/j.urology.2012.09.040.

86. Sfakianos, J.P., S.F. Shariat, R.L. Favaretto, et al., *Impact of smoking on outcomes after intravesical bacillus Calmette-Guerin therapy for urothelial carcinoma not invading muscle of the bladder.* BJU Int, 2011. **108**(4): p. 526-30. DOI: 10.1111/j.1464-410X.2010.09874.x.

87. Singla, N., A.Q. Haddad, N.M. Passoni, et al., *Anti-inflammatory use may not negatively impact oncologic outcomes following intravesical BCG for high-grade non-muscle-invasive bladder cancer.* World J Urol, 2017. **35**(1): p. 105-111. DOI: 10.1007/s00345-016-1853-z.

88. Skolarus, T.A., E.W. Lee, K.S. Virgo, et al., *Intravesical bacille Calmette-Guerin therapy for non-muscle-invasive bladder cancer: effects of concurrent statin therapy.* J Am Coll Surg, 2009. **209**(2): p. 248-53. DOI: 10.1016/j.jamcollsurg.2009.04.014.

89. Studer, U.E., S. Jenzer, C. Biedermann, et al., *Adjuvant treatment with a vitamin A analogue (etretinate) after transurethral resection of superficial bladder tumors. Final analysis of a prospective, randomized multicenter trial in Switzerland.* Eur Urol, 1995. **28**(4): p. 284-90.

90. Takashi, M., T. Murase, S. Mizuno, et al., *Multivariate evaluation of prognostic determinants in bladder cancer patients.* Urol Int, 1987. **42**(5): p. 368-74. DOI: 10.1159/000281996.

91. Thompson, I.M., M. Peek, and F.R. Rodriguez, *The impact of cigarette smoking on stage, grade and number of recurrences of transitional cell carcinoma of the bladder.* J Urol, 1987. **137**(3): p. 401-3.

92. Tang, L., G.R. Zirpoli, K. Guru, et al., *Intake of cruciferous vegetables modifies bladder cancer survival.* Cancer epidemiology, biomarkers & prevention : a publication of the American Association for Cancer Research, cosponsored by the American Society of Preventive Oncology, 2010. **19**(7): p. 1806-1811. DOI: 10.1158/1055-9965.EPI-10-0008.

93. Tu, H., C.P. Dinney, Y. Ye, et al., *Is folic acid safe for non-muscle-invasive bladder cancer patients? An evidence-based cohort study.* Am J Clin Nutr, 2018. **107**(2): p. 208-216. DOI: 10.1093/ajcn/nqx019.

94. van Osch, F.H., S.H. Jochems, F.J. van Schooten, et al., *Significant Role of Lifetime Cigarette Smoking in Worsening Bladder Cancer and Upper Tract Urothelial Carcinoma Prognosis: A Meta-Analysis.* J Urol, 2016. **195**(4 Pt 1): p. 872-9. DOI: 10.1016/j.juro.2015.10.139.

95. van Osch, F.H.M., S.H.J. Jochems, R.C. Reulen, et al., *The association between smoking cessation before and after diagnosis and non-muscle-invasive bladder cancer recurrence: a prospective cohort study.* Cancer Causes Control, 2018. **29**(7): p. 675-683. DOI: 10.1007/s10552-018-1046-8.

96. Wakai, K., Y. Ohno, K. Obata, et al., *Prognostic significance of selected lifestyle factors in urinary bladder cancer.* Japanese journal of cancer research : Gann, 1993. **84**(12): p. 1223-1229. DOI: 10.1111/j.1349-7006.1993.tb02826.x.

97. Westhoff, E., X. Wu, L.A. Kiemeney, et al., *Dietary patterns and risk of recurrence and progression in non-muscle-invasive bladder cancer.* Int J Cancer, 2018. **142**(9): p. 1797-1804. DOI: 10.1002/ijc.31214.

98. Westhoff, E., J.A. Witjes, N.E. Fleshner, et al., *Body Mass Index, Diet-Related Factors, and Bladder Cancer Prognosis: A Systematic Review and Meta-Analysis.* Bladder cancer (Amsterdam, Netherlands), 2018. **4**(1): p. 91-112. DOI: 10.3233/BLC-170147.

99. Witjes, J.A., A.P. vd Meijden, W. Doesburg, et al., *Influence of fibrin clot inhibitors on the efficacy of intravesical Bacillus Calmette-Guerin in the treatment of superficial bladder cancer. The Dutch Southeast Cooperative Urological Group.* Eur Urol, 1993. **23**(3): p. 366-70.

100. Wyszynski, A., S.A. Tanyos, J.R. Rees, et al., *Body mass and smoking are modifiable risk factors for recurrent bladder cancer.* Cancer, 2014. **120**(3): p. 408-14. DOI: 10.1002/cncr.28394.

101. Xu, T., Z. Zhu, X. Wang, et al., *Impact of body mass on recurrence and progression in Chinese patients with Ta, T1 urothelial bladder cancer.* Int Urol Nephrol, 2015. **47**(7): p. 1135-41. DOI: 10.1007/s11255-015-1013-1.

102. Yafi, F.A., A.G. Aprikian, J.L. Chin, et al., *Contemporary outcomes of 2287 patients with bladder cancer who were treated with radical cystectomy: a Canadian multicentre experience.* BJU Int, 2011. **108**(4): p. 539-45. DOI: 10.1111/j.1464-410X.2010.09912.x.

103. Yonekura, S., F. Terauchi, K. Hoshi, et al., *Optimal body mass index cut-point for predicting recurrence-free survival in patients with non-muscle-invasive urothelial carcinoma of bladder.* Oncol Lett, 2018. **16**(3): p. 4049-4056. DOI: 10.3892/ol.2018.9068.

104. Yu, G.P., J.S. Ostroff, Z.F. Zhang, et al., *Smoking history and cancer patient survival: a hospital cancer registry study.* Cancer Detect Prev, 1997. **21**(6): p. 497-509.

105. Yuruk, E., M. Tuken, A. Colakerol, et al., *The awareness of patients with non - muscle invasive bladder cancer regarding the importance of smoking cessation and their access to smoking cessation programs.* International braz j urol : official journal of the Brazilian Society of Urology, 2017. **43**(4): p. 607-614. DOI: 10.1590/S1677-5538.IBJU.2016.0014.
